# Human osteoarthritic articular cartilage stem cells suppress osteoclasts and improve subchondral bone remodeling in experimental knee osteoarthritis partially by releasing TNFAIP3

**DOI:** 10.1186/s13287-023-03411-7

**Published:** 2023-09-27

**Authors:** Zhi-Ling Li, Xiao-Tong Li, Rui-Cong Hao, Fei-Yan Wang, Yu-Xing Wang, Zhi-Dong Zhao, Pei-Lin Li, Bo-Feng Yin, Ning Mao, Li Ding, Heng Zhu

**Affiliations:** 1 Department of Stem Cells and Regenerative Medicine, Beijing Institute of Radiation Medicine, Road Taiping 27, Beijing, 100850 People’s Republic of China; 2 Basic Medical College of Anhui Medical University, Hefei, 230032 Anhui Province People’s Republic of China; 3 People’s Liberation Army General Hospital, Road Fuxing 28, Beijing, 100853 People’s Republic of China; 4 Beijing Institute of Basic Medical Sciences, Road Taiping 27, Beijing, 100850 People’s Republic of China; 5 Air Force Medical Center, PLA, Road Fucheng 30, Beijing, 100142 People’s Republic of China

**Keywords:** Cell isolation, Articular cartilage stem cells, Osteoclasts, Subchondral bone remodeling, Diseased microenvironments

## Abstract

**Background:**

Though articular cartilage stem cell (ACSC)-based therapies have been demonstrated to be a promising option in the treatment of diseased joints, the wide variety of cell isolation, the unknown therapeutic targets, and the incomplete understanding of the interactions of ACSCs with diseased microenvironments have limited the applications of ACSCs.

**Methods:**

In this study, the human ACSCs have been isolated from osteoarthritic articular cartilage by advantage of selection of anatomical location, the migratory property of the cells, and the combination of traumatic injury, mechanical stimuli and enzymatic digestion. The protective effects of ACSC infusion into osteoarthritis (OA) rat knees on osteochondral tissues were evaluated using micro-CT and pathological analyses. Moreover, the regulation of ACSCs on osteoarthritic osteoclasts and the underlying mechanisms in vivo and in vitro were explored by RNA-sequencing, pathological analyses and functional gain and loss experiments. The one-way ANOVA was used in multiple group data analysis.

**Results:**

The ACSCs showed typical stem cell-like characteristics including colony formation and committed osteo-chondrogenic capacity. In addition, intra-articular injection into knee joints yielded significant improvement on the abnormal subchondral bone remodeling of osteoarthritic rats. Bioinformatic and functional analysis showed that ACSCs suppressed osteoarthritic osteoclasts formation, and inflammatory joint microenvironment augmented the inhibitory effects. Further explorations demonstrated that ACSC-derived tumor necrosis factor alpha-induced protein 3 (TNFAIP3) remarkably contributed to the inhibition on osteoarhtritic osteoclasts and the improvement of abnormal subchondral bone remodeling.

**Conclusion:**

In summary, we have reported an easy and reproducible human ACSC isolation strategy and revealed their effects on subchondral bone remodeling in OA rats by releasing TNFAIP3 and suppressing osteoclasts in a diseased microenvironment responsive manner.

**Graphical abstract:**

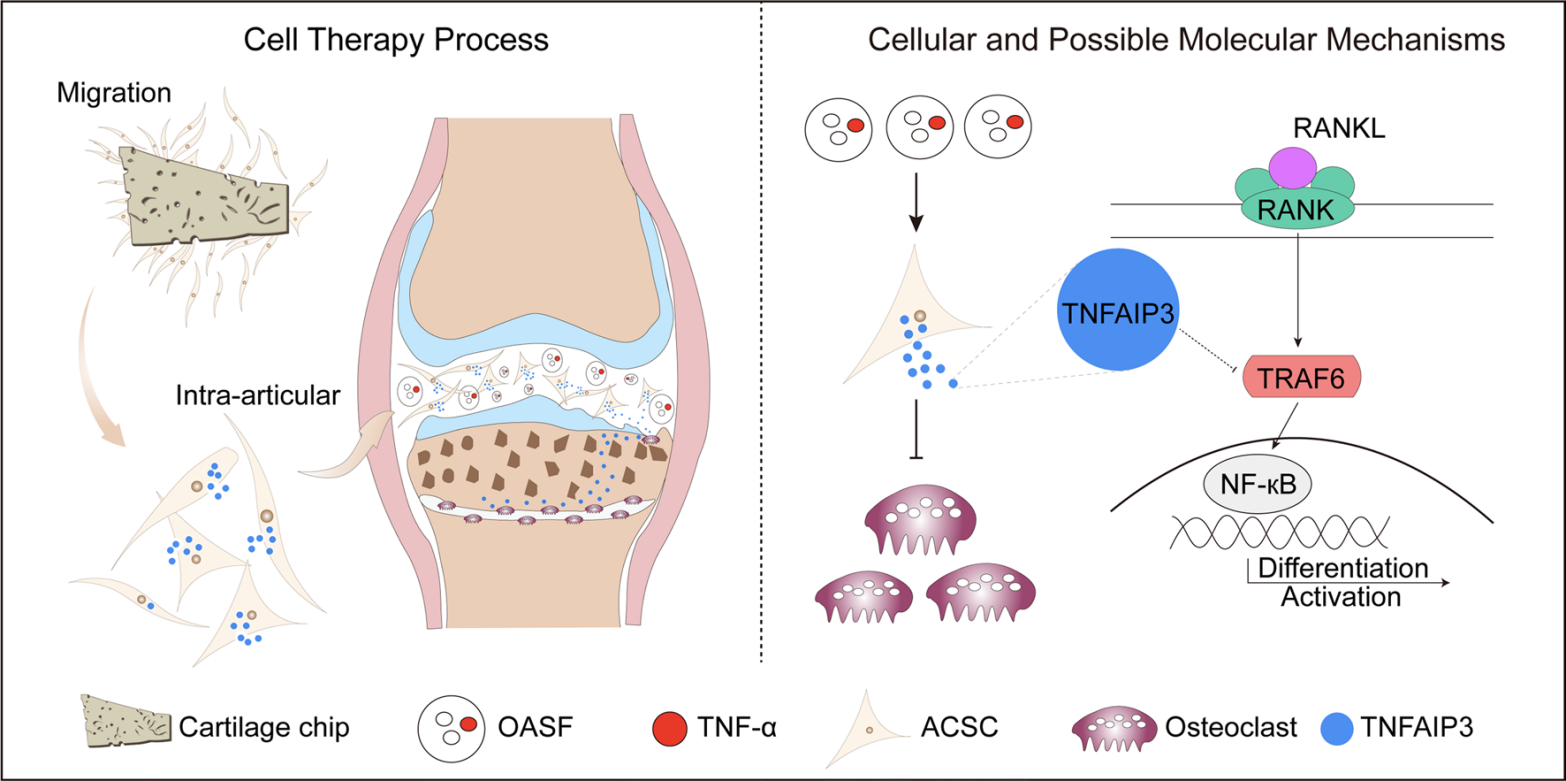

**Supplementary Information:**

The online version contains supplementary material available at 10.1186/s13287-023-03411-7.

## Introduction

Articular cartilage stem cells (ACSCs) have been identified as tissue-specific stem cells that reside in the articular cartilage and contribute to articular cartilage formation, homeostasis and regeneration [1–3]. ACSCs were originally isolated from bovine cartilage, and now have been identified from other species including rodent, rabbit, equine and human [1–3]. Under certain conditions, ACSCs can be clonally expanded and committedly differentiate into osteochondral tissues [1–3]. Thus, ACSCs have been used for the treatment of cartilage defects and tissue engineering, and seems superior to other types of stem cells [4–6]. However, the isolation procedures of ACSCs differ largely in previous studies, which results in the harvesting of heterogeneous cell populations [7, 8]. Additionally, the regenerative effects of ACSCs have been attributed to their osteo-chondrogenic potential but few data supports the engraftment of ACSCs and their descentant cells in vivo. Therefore, exploration of the unknown targets of ACSCs for the settlement of tissue regeneration is urgently needed. Moreover, the effects of in vivo microenvironments of tissue injuries and bone disorders on ACSCs and the underlying mechanisms remain to be investigated.

Osteoarthritis (OA) is originally characterized by the loss of articular cartilage, but now it is commonly accepted that all joint structures are affected [9, 10]. Though strategies for the settlement of OA keep evolving [11, 12], no available disease-modifying treatments are available to date partially due to the incomplete understanding of OA pathological progression. Recently, the role of subchondral bone remodeling in the pathogenesis of OA has gained increasing attention [13, 15, 16]. The increased subchondral bone resorption observed at the early stage of OA is mediated by osteoclasts. The generation of osteoclasts is highly dependent on microenvironments in which osteoclastogenesis-supporting cells, such as osteoblasts, osteocytes and activated T cells, express osteoclast-inductive factor, receptor activator of nuclear factor-κB ligand (RANKL). RANKL binds to its receptor RANK expressed by osteoclast precursors, and thereafter activates the signalling cascades, including mitogen-activated protein kinase (MAPK) and nuclear factor-κB (NF-κB) pathways via adaptor proteins such as tumor necrosis factor receptor-associated factors 6 (TRAF6) [14]. Mature osteoclasts adhere to the bone surface and secrete acid and lytic enzymes such as cathepsin K (CTSK) that degrade the bone matrix. Growing evidences have demonstrated that overactive osteoclasts and early-stage bone loss contribute to the histopathological alterations of subchondral bone remodeling, which plays a critical role in the initiation and progression of OA [13, 15, 16]. Data from previous studies proved that blocking osteoclastogenesis by drugs improved  the pathology in animal OA models, which suggested that osteoclasts may be one of the potential cellular targets for the treatment of OA [17].

Currently recommended clinical management of OA often includes oral and topical nonsteroidal anti-inflammatory drugs (NSAIDs) as first-line treatments, intra-articular hyaluronic acid (HA) injections to alleviate the pain, and surgical interventions like joint replacement surgery. However, there is no specific treatments that are able to slow down the progression of OA. Therefore, novel curative therapies for OA are urgently required [18]. In the past decades, stem cell therapy has been demonstrated to promote cartilage regeneration and alleviate OA evolution [19, 20]. Many studies have shown that mesenchymal stem cells (MSCs) have unique immunoregulatory properties, which exert profound anti-inflammatory effects via secreted immunoregulatory factors and contribute to tissue repair [21].

However, the impacts of diseased microenvironments on stem cell functions remains a major challenge to improve the efficacy of stem cell-based cartilage regeneration [22, 23]. During OA progression, inflammatory factors are known to control tissue remodeling and regeneration by influencing MSC differentiation [24]. Moreover, recipient tumor necrosis factor alpha (TNF-ɑ) have been proved to be able to elicit the immunosuppressive function of MSCs and facilitate tissue repair in inflammatory microenvironments [25, 26]. However, few data about the responses of ACSCs to OA microenvironments is available so far. Therefore, deciphering the impacts and mechanisms of critical diseased microenvironmental factors on ACSCs would be useful to promote both endogenous and exogenous ACSC-mediated tissue regeneration in OA. In previous studies, we have identified numerous stem/progenitor cells from bone and cartilage samples [27–31]. In addition, the cellular targets of these cells including osteoclasts, dendritic cells and macrophages have been determined, and the underlying mechanism of the regulations have been revealed [29, 30, 32].

By advantages of the easy and reproducible isolation protocol, we harvested human ACSCs and explored its effects in protecting knee osteo-chondrogenic tissue repair in OA rats in the present study. Moreover, the cellular and molecular mechanisms regarding the capacity of ACSCs to regulate subchondral bone remodeling in OA knees were also investigated.

## Materials and methods

### Ethics statement

All human tibial plateau specimens used in this study were collected from patients undergoing total knee arthroplasty surgery, with the approval of the Institutional Review Board at the Chinese People’s Liberation Army General Hospital (Beijing, China). Informed consent was obtained from all donors. Specific information is provided in Additional file 1: Table S1.

### Isolation and culture of primary hACSCs

Freshly collected human tibia plateau was rinsed in cold phosphate-buffered saline (PBS) (Servicebio, G4202) supplemented with 1% penicillin–streptomycin (Servicebio, G4005). Cartilage from the superficial layer of the lateral tibia plateau was cut into 1–2 mm slices using a sterile scalpel and washed in α-minimal essential Eagle’s medium (α-MEM) (Gibco, C12571500BT) containing 10% fetal bovine serum (FBS) (NEWZERUM, FBS-S500) and 1% penicillin–streptomycin. After minced with sterilized dissecting scissors, tissues were washed three times with PBS and transferred into 2-mL centrifuge tubes (Eppendorf, 0030 120.094) for enzymatic digestion (PBS containing 0.1% type II collagenase (Sigma-Aldrich, V900892) and 10% FBS) on a shaker at 80 r.p.m and 37 °C for 2 h. After removing released cells by centrifugation and washing with PBS, cartilage chips were seeded onto 25-cm^2^ tissue culture flasks (NEST, 707,001) (200 mg tissue per flask) and incubated at 37 °C, 5% CO_2_. After 7 days of culturing, fibroblast-like cells migrate out from cartilage chips (Additional file 2: Fig. 1A). After 14 days, cells reached confluency and were passaged into 75-cm^2^ tissue culture flasks. The medium (α-MEM containing 10% FBS) was changed every 3 days.

### Population doubling time (PDT) of hACSCs  

The proliferation kinetics of hACSCs was assessed by population doubling time (PDT) from P1 to P5 according to the formula:

$${\text{PDT}} = t \times [\lg 2/({\text{lgNt}} - {\text{lgN}}0)]$$

where N0 and Nt are the number of seeded and harvested cells, respectively, and t is the culture time (hours) [33].

### Trilineage differentiation and colony forming unit-fibroblast (CFU-F) assay

ACSCs (at passage 3 or 4) were cultured in adipogenic (Cyagen, GUXMX-90031), osteogenic (Cyagen, GUXMX-90021) and chondrogenic (Cyagen, GUXMX-90041) differentiation media according to the manufacturer’s instructions. Trilineage differentiation potentials were evaluated by staining of 0.5% Oil Red O (adipocytes), alkaline phosphatase (ALP) (osteoblasts) and 0.5% toluidine blue (chondrocytes). For CFU-F assays, ACSCs (at passage 3 or 4) were seeded into 6-well plate (500 cells/well) and incubated at 37 °C with 5% CO_2_. After 10 days, cells were fixed and stained with crystal violet staining solution.

### Animals

Seven-week-old female SD rats (*n* = 30) were purchased from Vital River Laboratory Animal Technology Co., Ltd (Beijing, China). Animals were randomly allocated to control and experimental groups, respectively. The rats were maintained under specific pathogen-free conditions, with ambient temperature (20–25 °C), humidity (45–65%) and light cycle (12 h light/dark).

### Flow cytometry

The following antibodies were used: CD45-PE-Cy7 (Biolegend, 304,016, 5 μL/test), CD31-PE-Cy7 (Biolegend, 303,118, 5 μL/test), CD235A-PE-Cy7 (Biolegend, 349,112, 5 μL/test), CD29-PE (eBioscience, 12-0299-41, 5 μL/test), CD44-APC (Invitrogen, 17–0441-81, 5 μL/test), CD90-FITC (Biolegend, 328,108, 5 μL/test), CD105-PE (Invitrogen, 1,930,337, 5 μL/test) and PDPN-APC (Biolegend, 337,022, 5 μL /test). ACSCs were stained in 100 μL PBS for 30 min at 4 °C, washed once and resuspended in PBS. Unstained cells were used as control. Flow cytometry was performed on BD FACS Aria II. The analyses was performed using Flowjo 8.0.

### Induction of post-traumatic osteoarthritis (PTOA) rat model and treatment

After 7 days of acclimation, rats were subjected to anterior cruciate ligament transection (ACLT) surgery to induce mechanical instability-associated OA on both knees as described previously with minor revisions [32]. Briefly, rats were anesthetized by intraperitoneally injection of 2% pentobarbital sodium (0.25 mL/100 g). Both knees of rats were shaved and disinfected for surgery. The sham operation was conducted by opening the joint capsule and then suturing the incision. For the ACLT group, the medial joint capsule adjacent to the patellar tendon was incised with a scalpel. After carefully cutting off the anterior cruciate ligament, the medial capsular incision was then closed with sutures.

At 1 week post-surgery, rats received intra-articular injections of 1 × 10^5^ or 1 × 10^6^ ACSCs in 100 μL PBS as described previously [32]. At 4 weeks after injection, rats were euthanized by CO2 asphyxiation and the whole knee joints were excised, with surrounding soft tissues removed, for micro-CT and histological analyses (*n* = 3).

### Micro-CT analysis

Rat knee joints were fixed with 4% paraformaldehyde for 48 h and scanned on a micro-CT scanner (Viva CT40, Scanco) at a resolution of 8 μm (55 kV, 114 mA, 500 ms integration time). 3D image reconstruction and visualization were performed using CTvox software (v.2.0.0). Data analyses was accomplished with CTAn software (v.1.18). The following parameters were determined: bone volume fraction (BV/TV), trabecular bone thickness (Tb.Th), trabecular bone number (Tb.N), and trabecular separation (Tb.Sp). The severity of osteoarthritic changes (OA grade) was graded through quantification of the degree of cartilage loss and osteophytes by Micro-CT as described before [34].

### Histology, immunohistochemistry and immunofluorescence

Rat knee joints were fixed with 4% paraformaldehyde for 48 h, decalcified with 10% EDTA (pH 7.2–7.4) for 4 weeks, then processed and embedded in paraffin. Afterwards, the samples were cut into 4 μm-thick sections and stained with safranin O–fast green (SOFG) and toluidine blue. Collagen II (Abcam, ab34712) and TRAP (Servicebio, G1050-50 T) were detected by immunohistochemistry. The expression levels of TRAF6 and CTSK were determined by immunofluorescence. Primary and secondary antibodies were used as follows: rabbit anti-Collagen II (Abcam, ab34712), rabbit anti-TRAF6 (Abcam, ab40675), rabbit anti-CTSK (Abcam, ab19027), HRP-labeled goat anti-rabbit IgG (Servicebio, GB23303) and Alexa Fluor® 488-labeled goat anti-rabbit IgG (Servicebio, GB25303). Inverted microscope (Olympus, CKX53) equipped with a cooled CMOS camera (Tucsen, FL-20BW) was used for image acquisition. Semi-quantitative analyses of immunohistochemistry and immunofluorescence staining were performed using ImageJ software (version 1.53) as described before [35].

### Total RNA extraction and high-throughput RNA sequencing

Total RNA of ACSCs treated with or without 20 ng/mL human TNF-α (PeproTech, 300-01A) (for 3 days) was extracted using TRI Reagent (Sigma-Aldrich, T9424) according to the manufacturer’s instructions. Total RNA of each sample was quantified and qualified by Agilent 2100/2200 Bioanalyzer (Agilent Technologies, Palo Alto, CA, USA), NanoDrop (Thermo Fisher Scientific Inc.). 1 μg total RNA was used for following library preparation. Next generation sequencing library preparations were constructed according to the manufacturer’s protocol. The poly(A) mRNA isolation was performed using Poly(A) mRNA Magnetic Isolation Module or rRNA removal Kit. The mRNA fragmentation and priming was performed using First Strand Synthesis Reaction Buffer and Random Primers. First strand cDNA was synthesized using ProtoScript II Reverse Transcriptase and the second-strand cDNA was synthesized using Second Strand Synthesis Enzyme Mix. Sequencing was carried out using a 2 × 150 paired-end (PE) configuration; image analysis and base calling were conducted by the HiSeq Control Software (HCS) + OLB + GAPipeline-1.6 (Illumina) on the HiSeq instrument, image analysis and base calling were conducted by the NovaSeq Control Software (NCS) + OLB + GAPipeline1.6 (Illumina) on the NovaSeq instrument, image analysis and base calling were conducted by the Zebeacall on the MGI2000 instrument.

### Bioinformatics analyses

Differential expression analyses were performed using Limma (v.3.46.0) and DESeq2 (v.1.30.1) packages. Gene ontology (GO) analyses and Gene Set Enrichment Analysis (GSEA) were performed with clusterProfiler (v.4.5.2). For data visualization, ggplot2 (v.3.3.6), ggvenn (v.0.1.9), gg.gap (v.1.3), ggforce (v.0.4.0), ggradar (v.0.2) and GseaVis (v.0.0.2) were used.

### Western blotting and enzyme-linked immunosorbent assay(ELISA)

Protein lysates were prepared using Laemmli buffer (Bio-Rad, 1610737) and then separated on SDS-PAGE gels. Following transfer, PVDF membranes were incubated with anti-GAPDH (Proteintech, 10,494-1-AP) and anti-TNFAIP3 (Abcam, ab92324). ACSCs were cultured in the presence of TNF-α for 72 h and TNFAIP3 concentrations in the culture supernatants were measured using human TNFAIP3 ELISA kit (CZKWbio, SU-BN14310) according to the manufacturer’s instructions. The concentration of TNF-ɑ within the OASF before and after neutralization by anti-human TNF-ɑ neutralizing antibody (BioLegend, 502,902) (250 ng/mL) was determined using TNF-ɑ ELISA kit (CZKWbio, AK-A11776) according to the manufacturer’s instructions.

### Lentiviral vector packaging and cell transfection

The human TNFAIP3-overexpress and TNFAIP3-knockdown lentivirus and lentiviral vector controls were obtained from Shanghai GenePharma Co., Ltd. Briefly, shRNA sequences were designed based on the gene sequence of TNFAIP3 and synthesized by GenePharma, then linked with lentivirus vector LV-3 or LV-5 to construct the recombined shRNA vectors of high-titer (~ 10^8^ TU/mL). Then the lentiviral vectors were efficiently transduced into ACSCs according to the instructions of the manufacturer. Infected cells were selected with 3 μg/mL puromycin for 3 days.

### In vitro generation of osteoclasts from human peripheral blood mononuclear cells (PBMCs)

The research on human PBMC samples involved in this work was approved by the Research Ethics Committee of Military Medical Sciences, PLA. The initial Ethics approval (AF/SC-08102.83) was obtained on March 9, 2021. Human peripheral blood mononuclear cells (PBMCs) were separated from whole blood of healthy volunteers (*n* = 12) by density gradient centrifugation using lymphocyte separation medium (TBD, LTS1077) according to the manufacturer’s protocol. After cultured overnight in α-MEM supplemented with 10% FBS and 1% penicillin–streptomycin, floating cells in the supernatant were discarded. Round adherent cells were collected by gentle PBS washing and centrifugation. Afterwards, primary adherent mononuclear cells were seeded in 48-well culture plates (1 × 10^5^ cells per well) and maintained in α-MEM supplemented with 10% FBS, 20 ng/mL human Microphage colony stimulating factor (M-CSF) (PeproTech, 30,025) and 20 ng/mL human RANKL (PeproTech, 310-01) for 9 days [29]. Untreated adherent mononuclear cells were set as the control group.

To investigate the effects of ACSCs on osteoclasts, osteoclasts were co-cultured with 5 × 10^3^ ACSCs or the culture supernatant of ACSCs (5% or 10%) in a Transwell co-culture system or 48-well culture plates.

Synovial fluid from OA patients (OASF) was harvested from the knee joints of OA patients (*n* = 10) as previously described [32] (Additional file 3: Table S2). Informed consent was obtained from all volunteers and ethics approval was obtained from the PLA General Hospital Research Ethics Committee.

In some groups, OASF (5%) [32], human recombinant TNF-α (20 ng/mL), anti-human TNF-α neutralizing antibody (BioLegend, 502,902) (250 ng/mL), human recombinant TNFAIP3 (Abcam, ab271357) (20 ng/mL), and anti-TNFAIP3 neutralizing antibody (GeneTex, GTX31376) (100 ng/mL) were added into the co-culture system of corresponding groups.

### Bone resorption pit assay

To assess bone resorption activity of osteoclasts, we performed pit assay on sterile elephant tusk slices prepared from discarded elephant tusks (Dentine Discs, IDS). Tusk slices (diameter: 5 mm; thickness: 0.3 mm) were washed three times with culture medium before they were carefully placed at the bottom of the wells of a 48-well culture plate. ACSCs were seeded onto each slice at a density of 5 × 10^3^ per well and incubated for 24 h, followed by the addition of adherent mononuclear cells (1 × 10^5^ per well), and cultured in the presence of 20 ng/mL human M-CSF and 20 ng/mL human RANKL for 3 weeks. Cell-free tusk slices were stained with Toluidine blue to detect resorption pits under microscope.

### TRAP staining

Cells were fixed and stained for TRAP using an leukocyte acid phosphatase kit (Sigma-Aldrich, 386A-1KT) according to the manufacturer’s instructions. The number of large TRAP-positive multinucleated (≥ 3 nuclei) cells from each group was counted for statistical analysis.

### Statistical analysis

All statistical analyses were performed using GraphPad Prism software (version 8.0.2). Single comparisons and multiple comparisons were analyzed using Student’s t test and one-way ANOVA, respectively. *P* < 0.05 was considered statistically significant (*P < 0.05; **P < 0.01; ***P < 0.001).

## Results

### Isolation of ACSCs from human tibial plateau cartilage and functional assays

**Fig. 1:**
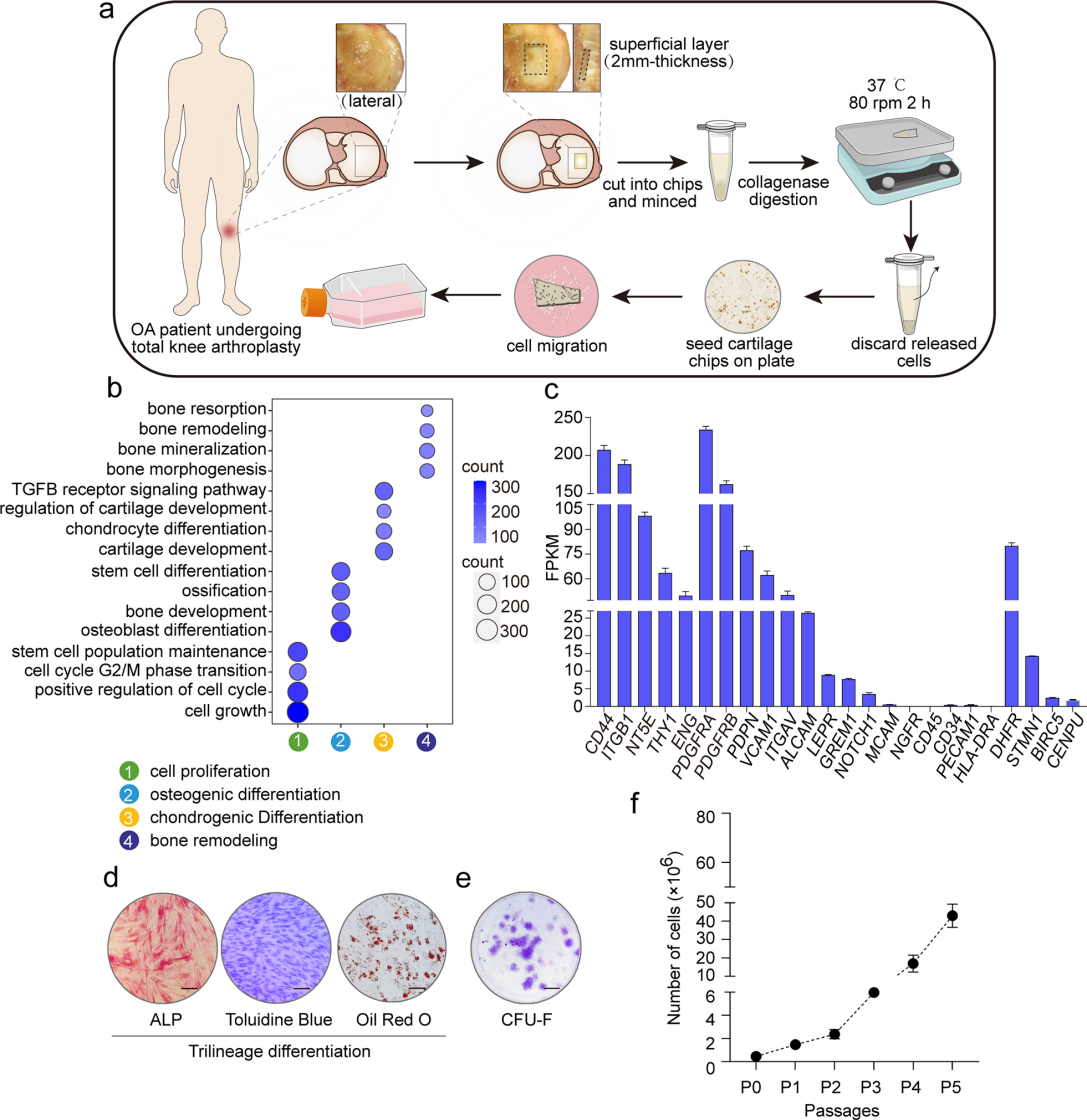
Isolation and characterization of ACSCs. **a** Graphical illustration of the isolation workflow. Human tibial cartilage slices were minced, enzymatically digested and seeded onto tissue culture flasks. After cultured for 1 week, ACSCs migrated out of cartilage chips. The images in Fig. 1a are depicted by Zhi-Ling Li. **b** Dot plots showing enriched Gene Ontology (GO) terms in ACSCs associated with proliferation, osteo-chondro differentiation, and bone remodeling. **c** Bar plot showing the expression level of related MSC surface markers in ACSCs (*n* = 3). **d** Trilineage differentiation abilities of ACSCs were examined via Alkaline phosphatase (left; osteoblasts), Toluidine blue (middle; chondrocytes) and Oil red O (right; adipocytes) staining. No significant difference were identified for the trilineage differentiation ability of ACSCs from passage 2 to passage 6. Scale bars, 200 μm. **e** Representative crystal violet staining for the colony formation ability of ACSCs (passage 3–4 were used). Scale bar, 2 mm. **f** Proliferation curves of ACSCs from primary culture to passage 5 (*n* = 3)

We applied a reproducible method to isolate primary ACSCs from human tibial plateau cartilage (Fig. 1a and Methods). At 7 days after the initial seeding, fibroblast-like cells migrated out from the articular cartilage chips and formed an adherent layer (Additional file 2: Fig. S1a). After in vitro expansion for 2 weeks, the initial adherent spindle-shaped cells developed into a more confluent monolayer comprised of morphologically homogenous fibroblast-like cells (Additional file 2: Fig. S1b). Our RNA-seq data showed that human ACSCs were highly enriched for Gene Ontology (GO) terms related to self-renewal, proliferation, multiple differentiation and bone remodeling (Fig. 1b). In addition, ACSCs did not express hematopoietic and endothelial markers (*CD34*, *CD45*, *HLA-DR* and *CD31*) and were positive for putative MSC markers (such as *CD44*, *ITGB1*, *NT5E*, *THY1* and *ENG*) and previously described interstitial markers including *PDGFRA*, *PDGFRB* and *VCAM1*, as well as newly discovered stem cell markers like *PDPN*, *DHFR* and *STMN1* etc. (Fig. 1c and Additional file 2: Fig. S1c). Further functional assays showed that hACSCs committed strongly to osteoblasts and chondrocytes (Fig. 1d). Besides, hACSCs were capable of growing into large colonies (Fig. 1e). Quantitative analysis of cell yields showed that an average of 7.5 × 10^6^ hACSCs per gram cartilage chips were obtained with this protocol in 2 weeks (Fig. 1f). The population doubling time (PDT) of hACSCs from P1 to P5 was also assessed (Additional file 4: Fig. S2).

### Administration of hACSCs alleviates knee osteo-chondral lesions in surgically induced OA rats

**Fig. 2:**
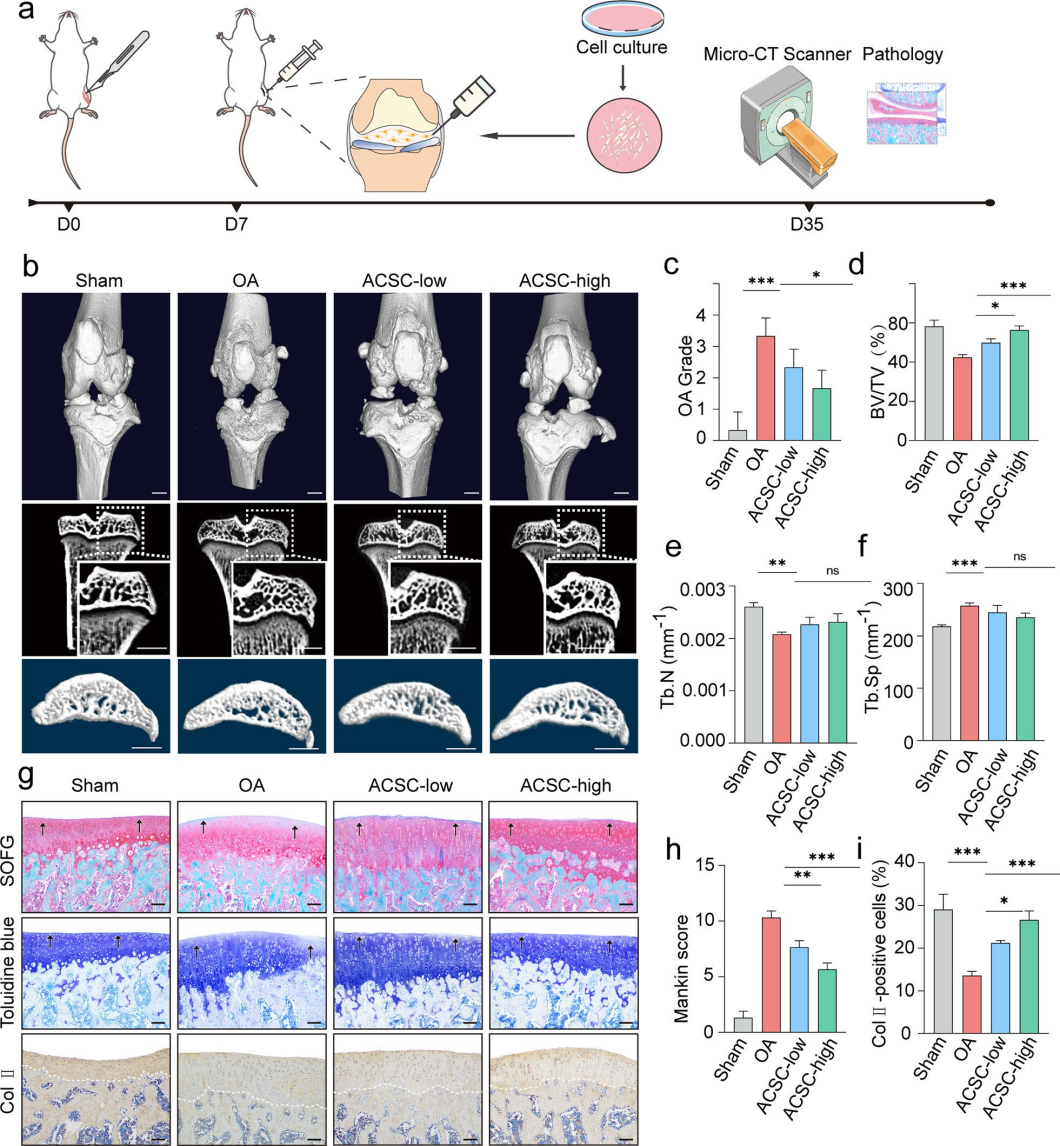
Intra-articular ACSCs injection ameliorates surgery-induced OA in rats. **a** Schematic of the experimental design. Rats were subjected to ACLT surgery and received intra-articular ACSCs injection (1 × 10^5^ or 1 × 10^6^ cells) for treatment at 1 w post-surgery (*n* = 3). **b** 3D reconstruction of rat knee joints (top), tibial subchondral bone medial compartment (sagittal view) (bottom) and microCT images of rat tibia subchondral bone (coronal view) (middle) showed that ACSCs significantly improved the osteochondral structure of OA rats at 4 week after injection. **c** OA grade was rated by quantification of the degree of cartilage loss and osteophytes according to micro-CT. **d**–**f** Micro-CT analysis of bone volume fraction (BV/TV) (d), trabecular number (Tb.N) (e) and trabecular separation (Tb.Sp) (f) in the tibial subchondral bone of OA rats. **g** Safranin O–fast green staining (top), toluidine blue staining (middle), and type II collagen immunostaining (bottom) showed that ACSCs significantly improved the pathological changes in the osteochondral regions of OA rats. Scale bars, 200 μm. **h** Mankin scores of osteoarthritic rat knee articular cartilage at 4 weeks after injection. High-ACSCs group had significantly lower pathological scores than the OA groups. **i** Quantification of Col II-positive cells in **(g)**. The statistical significance of differences was determined using one-way ANOVA with multiple comparison tests (LSD). Experiments were repeated independently for more than three times. All data are shown as the mean ± S.D. *P < 0.05, **P < 0.01, ***P < 0.001; ns, not significant

To further investigate the potential therapeutic effects of ACSCs, we induced OA in 8-week-old female SD rats and performed intra-articular injection of hACSCs after 1 w post-surgery (Fig. 2a). Micro-CT scanning revealed that ACSCs treatment significantly alleviated joint destruction and abnormal subchondral bone remodeling in OA rat knees at 4 weeks post-injection (Fig. 2b). Quantitative micro-CT analyses revealed that ACSCs injection alleviated the grade of OA, loss of bone volume and abnormal bone remodeling in OA rats (Fig. 2c-f). In addition, the protective effects were strengthened with the increase of ACSCs. The histological analysis is consistent with the imaging results (Fig. 2g). Briefly, Safranin O–fast green staining showed that ACSCs alleviated the osteochondral erosion of OA rat knees. Toluidine blue and immunohistochemistry staining for type II Collagen showed that ACSCs-mediated improvement of osteo-chondral structure is associated with extracelluar matrix homeostasis (Fig. 2g) and decreased Mankin scores (Fig. 2h). The quntitative analysis data of Collagen II staining is consistent with the graphical observation (Fig. 2i).

### ACSCs inhibit OA osteoclast formation in vitro and in vivo

**Fig. 3:**
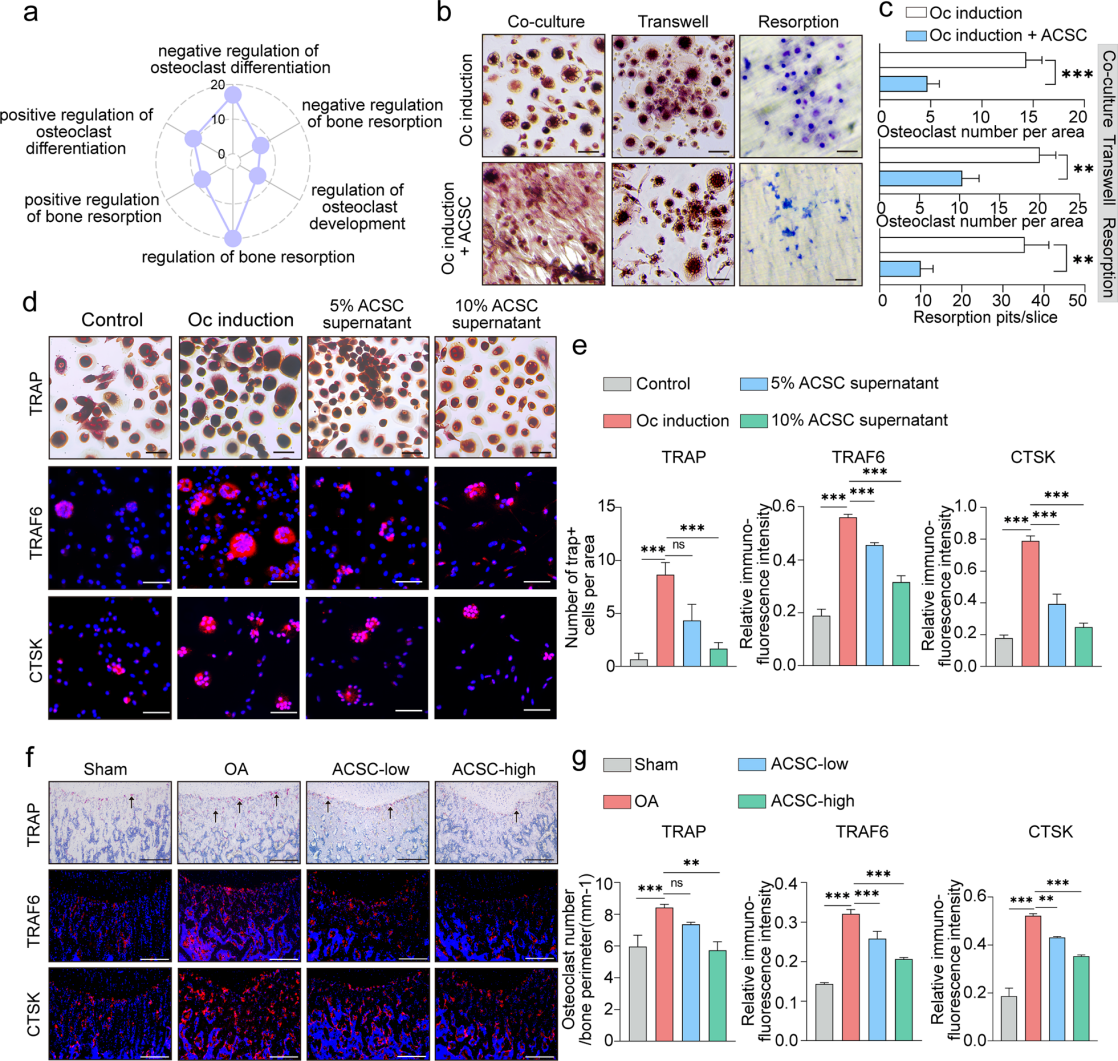
ACSCs inhibit osteoclastogenesis in vitro and in vivo. **a** Radar plot showing enriched osteoclast-related GO terms in ACSCs. Each axis starts at 0 and ends at 20. **b** TRAP staining of osteoclasts co-cultured (left) without or with ACSCs in transwell plates (middle) and toluidine blue staining for resorption pits (right) showed that ACSCs inhibit OC formation via secreted factors. Scale bars, 50 μm. **c** Quantification of TRAP^+^ multinucleated osteoclasts (> 3 nuclei) (top and middle) and resorption pits (bottom) in (b). **d** TRAP staining (top) and immunofluorescence staining of TRAF6 (middle) and CTSK (bottom) showed that the culture supernatant of ACSCs (5% or 10%) inhibits OC formation and OC differentiation-related gene expression. Scale bars, 50 μm. **e** Quantification of TRAP^+^ multinucleated osteoclasts (> 3 nuclei) (left) and the relative fluorescence intensity of TRAF6 (middle) and CTSK (right) in (d). **f** TRAP staining (top) and immunofluorescence of TRAF6 (middle) and CTSK (bottom) (genes associated with OC formation and OC differentiation) in the subchondral bone of OA rats at 4 week after injection (*n* = 3). Scale bars, 200 μm. **g** Quantification of osteoclast number (left) and the relative fluorescence intensity of TRAF6 (middle) and CTSK (right) in (f). The statistical significance of differences was determined using one-way ANOVA with multiple comparison tests (LSD). Experiments were repeated independently for more than three times. All data are shown as the mean ± S.D. **P < 0.01, ***P < 0.001; ns, not significant

Since aberrant subchondral bone remodeling is a major pathological change of OA and osteoclast activation is involved in the subchondral bone remodeling in early-stage OA, we next investigated the possible role of ACSCs in modulating osteoclast formation during OA progression [15]. RNA sequencing analysis indicated that ACSCs were also enriched for osteoclast-related pathways (Fig. 3a). To investigate the effects of ACSCs on OA osteolcast formation, we co-cultured osteoclasts with ACSCs in a Transwell system that allows the transfer of soluble factors. The results of tartrate resistant acid phosphatase (TRAP) staining showed that ACSCs significantly suppressed multinucleated osteoclast formation without cellular contact (Fig. 3b, c). Moreover, pit formation assay on dentine slices demonstrated that osteoclasts produced fewer resorption pits in the presence of ACSCs (Fig. 3b, c). Further analysis of TRAP staining and immunofluorescence staining of TRAF6 and CTSK showed that ACSC conditional medium inhibited osteoclast formation in a dose dependent manner (Fig. 3d, e). Furthermore, the in vivo data showed that ACSCs injection remarkably reduced the number of TRAP^+^ cells, and cells that expressing TRAF6 and CTSK in the subchondral area of OA rat knees. Importantly, the inhibitory effect was ACSC cell number dependent (Fig. 3f, g).

### ACSCs inhibit osteoclast formation in inflammatory microenvironment

**Fig. 4:**
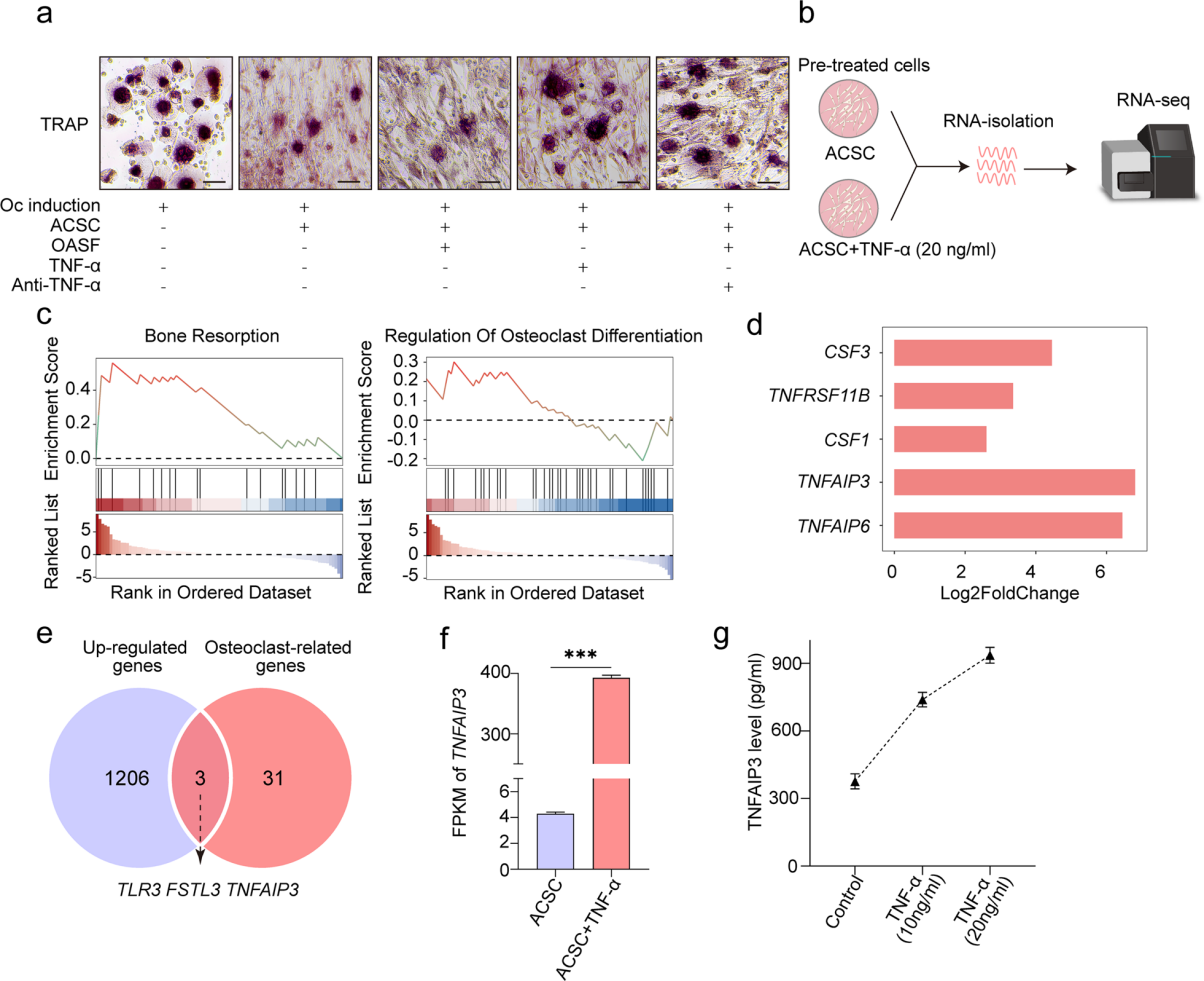
The function and expression of TNFAIP3 in ACSCs. **a** Representative TRAP staining images of osteoclasts during in vitro osteoclast formation in the presence of 5% OASF, 20 ng/mL TNF-α or 250 ng/mL anti-human TNF-α neutralizing antibody showing that TNF-α and OASF (containing TNF-α) suppress osteoclast formation. Scale bar, 50 μm. **b** Schematic overview of the RNA sequencing workflow. ACSCs were treated with 20 ng/mL TNF-α for 3 d, followed by RNA isolation, cDNA library construction and high-throughput sequencing (*n* = 3). **c** GSEA enrichment plots showed that ACSCs were more actively involved in osteoclast-related biological processes after TNF-α stimulation. **d** Bar plot showing the differential expression of osteoclast-related genes upon TNF-α stimulation. **e** Venn diagram indicated upregulated osteoclast-related DEGs in TNF-α stimulated ACSCs. **f** Gene expression of *TNFAIP3* in ACSCs treated without or with 20 ng/mL TNF-α. **g** ELISA quantification of TNFAIP3 in the culture supernatant of ACSCs treated without or with TNF-α (10 ng/mL or 20 ng/mL) for 3 days (*n* = 3)

To investigate the influence of inflammatory microenvironment on the therapeutic effects of ACSCs, ACSCs and osteoclasts were co-cultured in the presence of OASF. TRAP staining data showed the OASF strengthened the suppressive effects of ACSCs on osteoclast formation in vitro. The concentration of TNF-ɑ within the OASF before and after neutralization has been included in Additional file 5: Table S3. The addition of anti-TNF-ɑ neutralization antibody into the co-culture system partially abolished the promoting of OASF, which suggest that ACSCs respond to the diseased microenvironments, and inflammatory cytokines including TNF-ɑ were closely involved in the alternative regulation (Fig. 4a).

### ACSCs inhibit OA osteoclasts partially via secreting TNFAIP3

**Fig. 5:**
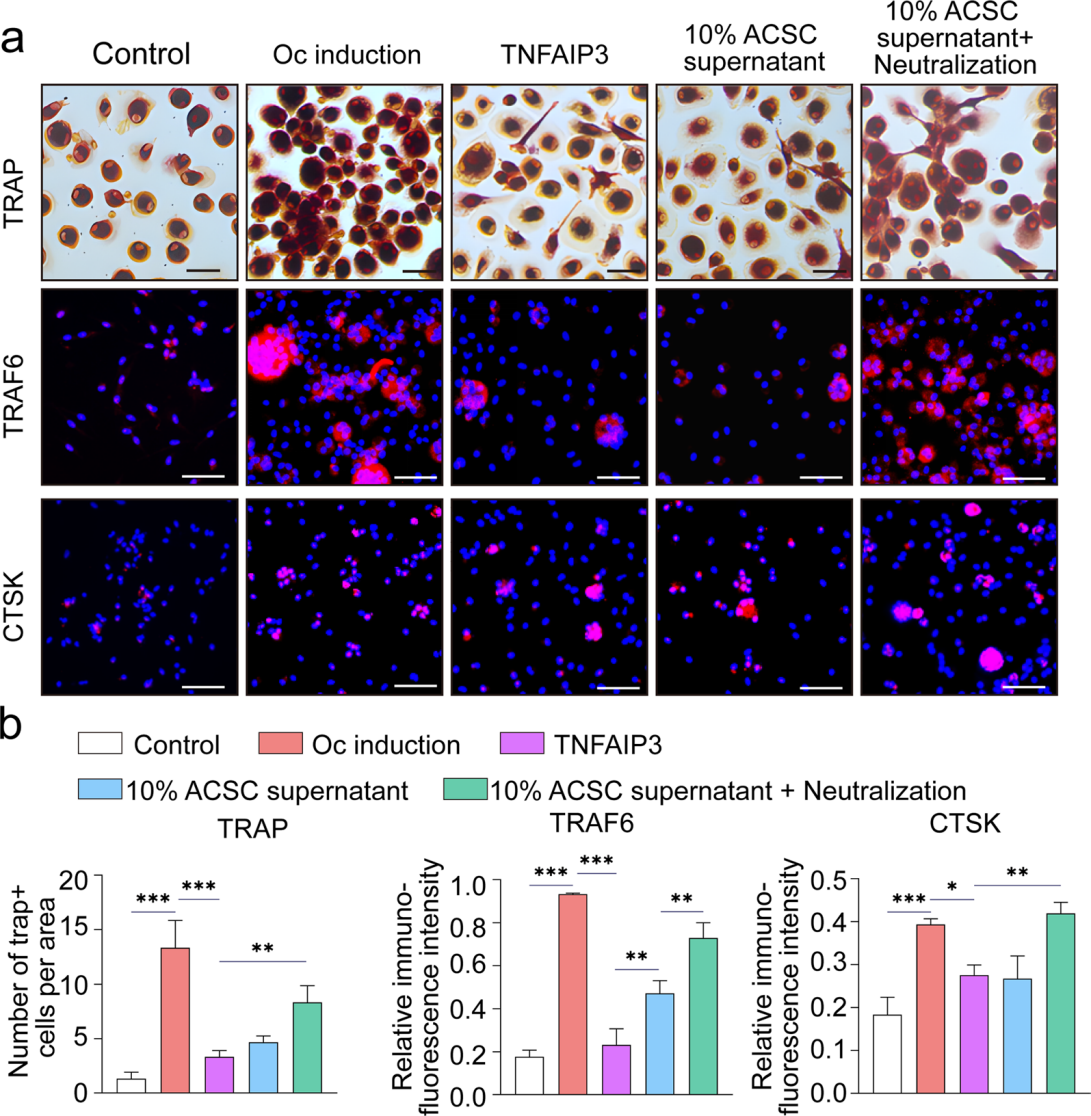
ACSC-derived TNFAIP3 suppresses osteoclastogenesis in vitro. **a** TRAP staining (top) and immunofluorescence staining of TRAF6 (middle) and CTSK (bottom) showed that the culture supernatant of ACSCs (10%) and soluble TNFAIP3 protein (20 ng/mL) inhibit OC formation and OC differentiation-related gene expression, while addition of the anti-TNFAIP3 neutralizing antibody (100 ng/mL) promotes OC formation. Scale bars, 50 μm. **b** Quantification of TRAP^+^ multinucleated osteoclasts (> 3 nuclei) (left) and relative fluorescence intensity of TRAF6 (middle) and CTSK (right) in **(a)**. The statistical significance of differences was determined using one-way ANOVA with multiple comparison tests (LSD). Experiments were repeated independently for more than three times. All data are shown as the mean ± S.D. **P < 0.01, ***P < 0.001; ns, not significant

**Fig. 6:**
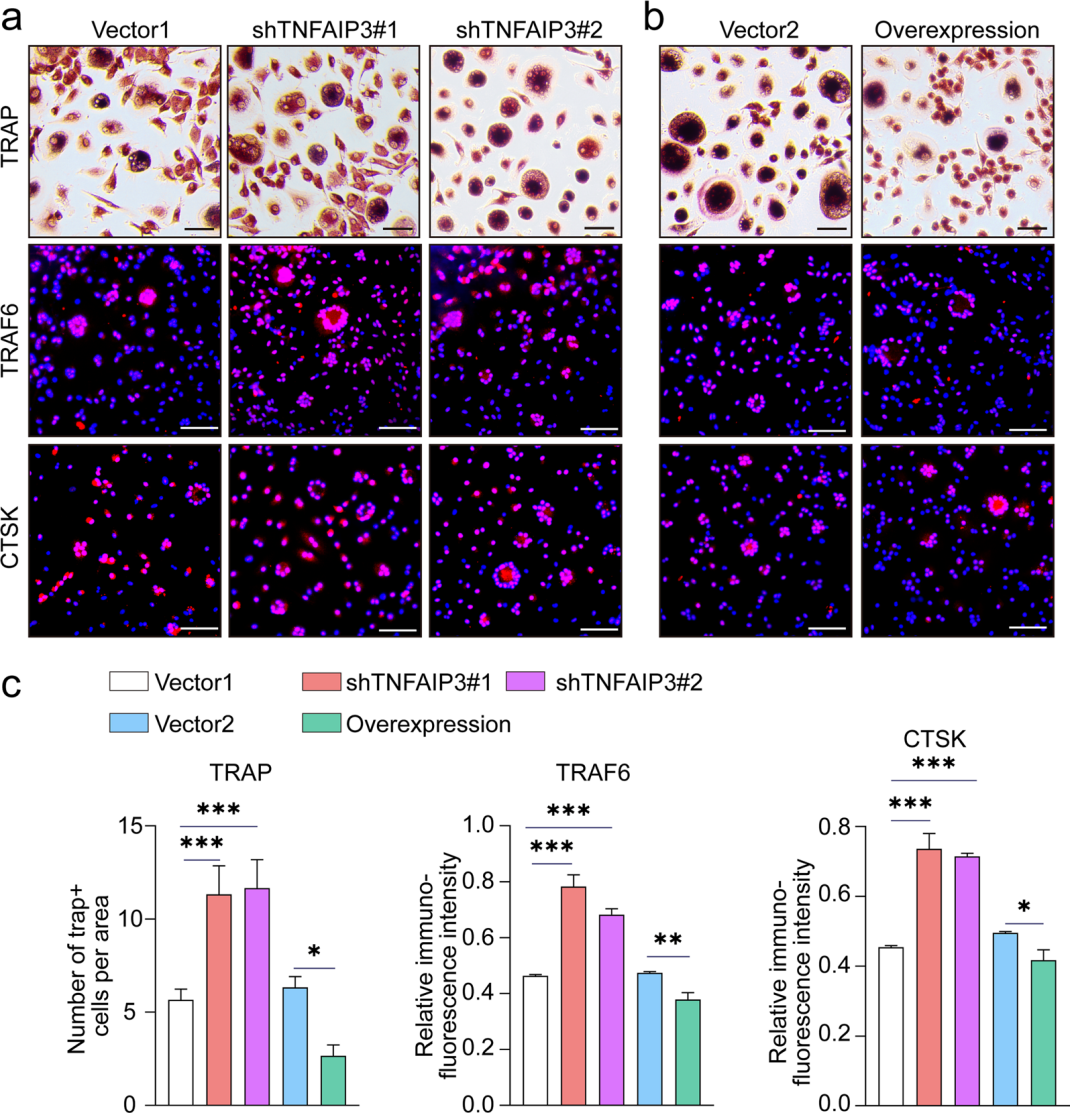
Overexpression of TNFAIP3 in ACSCs inhibits osteoclastogenesis in vitro. **a**, **b** TRAP staining (top) and immunofluorescence of TRAF6 (middle) and CTSK (bottom) in osteoclasts treated with 10% culture supernatant of transfected ACSCs. Knockdown of TNFAIP3 promotes OC formation, while overexpression of TNFAIP3 inhibits OC formation. Scale bars, 50 μm. **c** Quantification of TRAP^+^ multinucleated osteoclasts (> 3 nuclei) (left) and the relative fluorescence intensity of TRAF6 (middle) and CTSK (right) in **(a**–**b)**. The statistical significance of differences was determined using one-way ANOVA with multiple comparison tests (LSD). Experiments were repeated independently for more than three times. All data are shown as the mean ± S.D. *P < 0.05, **P < 0.01, ***P < 0.001

Based on the result that TNF-ɑ in OASF enhanced the suppressive effects of ACSCs on osteoclasts, high-throughput RNA sequencing of TNF-ɑ primed ACSCs was performed (Fig. 4b). GSEA analyses indicated that ACSCs were more associated with osteoclast-related biological processes upon TNF-ɑ stimulation (Fig. 4c). The bioinformatics data showed that the expression of *TNFAIP3*, TNF receptor superfamily member 11b (*TNFRSF11B*) and *TSG6* in ACSCs significantly increased (Fig. 4d). Venn plot indicated up-regulated DEGs in TNF-ɑ-stimulated ACSCs that are associated with osteoclast-related biological process and led to *TNFAIP3* (Fig. 4e). The gene expression level of *TNFAIP3* increased upon TNF-ɑ stimulation (Fig. 4f). Further ELISA data proved the increase of secretory TNFAIP3 from ACSCs in the presence of TNF-ɑ (Fig. 4g). In addition, the results of osteoclast formation assays showed that the addition of TNFAIP3 exerted a significant suppression on osteoclast formation in *vitro* (Fig. 5a, b). Moreover, blockage of TNFAIP3 in ACSC conditional medium partially abolished the suppressive effects on osteoclast formation. Furthermore, the conditional medium from TNFAIP3 over-expressing ACSCs or TNFAIP3 deficient ACSCs exhibit strengthened or weakened suppressive effects on osteoclasts in vitro (Fig. 6a–c).

### TNFAIP3 modification modulated the protecting effects of ACSCs on OA subchondral bone remodeling by targeting osteoclasts in situ

**Fig. 7:**
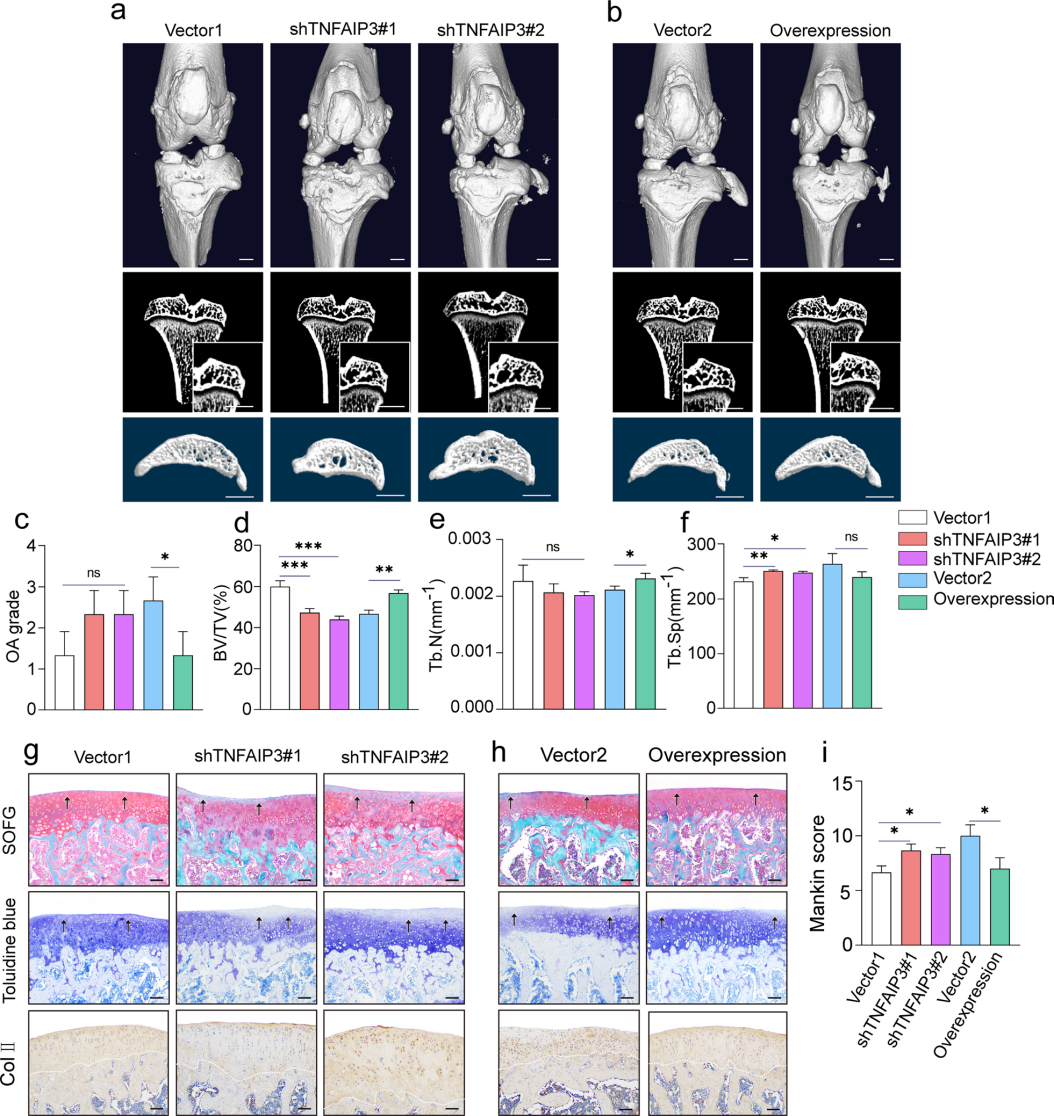
ACSC-specific TNFAIP3 overexpression ameliorates surgery-induced OA in rats. **a**, **b** 3D reconstruction of rat knee joints (top), tibial subchondral bone medial compartment (sagittal view) (bottom), and microCT images of rat tibia subchondral bone (coronal view) (middle) showed that TNFAIP3-overexpression ACSCs significantly improved the osteochondral structure, while TNFAIP3-knockdown ACSCs lead to poor protection effects in OA rats at 4 week after injection (*n* = 3). Scale bars, 2 mm. **c** OA grade was rated by quantification of the degree of cartilage loss and osteophytes from micro-CT. **d**–**f** Micro-CT measurements for bone volume fraction (BV/TV) **(d)**, trabecular number (Tb.N) **(e)** and trabecular separation (Tb.Sp) **(f)** in the subchondral bone of vector-transfected and TNFAIP3-overexpress or knockdown ACSCs rats at 4 w after injection. **g**, **h** Representative images of safranin O–fast green staining (top), toluidine blue staining (middle), and type II collagen immunostaining (bottom) in the subchondral bone showed that TNFAIP3-overexpression ACSCs significantly improved the pathological changes in the osteochondral regions of OA rats, while TNFAIP3-knockdown ACSCs lead to poor effects in OA models at 4 w post-surgery. Scale bars, 200 μm. **i** Mankin scores of osteoarthritic rat knees at 4 w after injection. The statistical significance of differences was determined using one-way ANOVA with multiple comparison tests (LSD). All data are shown as the mean ± S.D. *P < 0.05, **P < 0.01, ***P < 0.001; ns, not significant

**Fig. 8:**
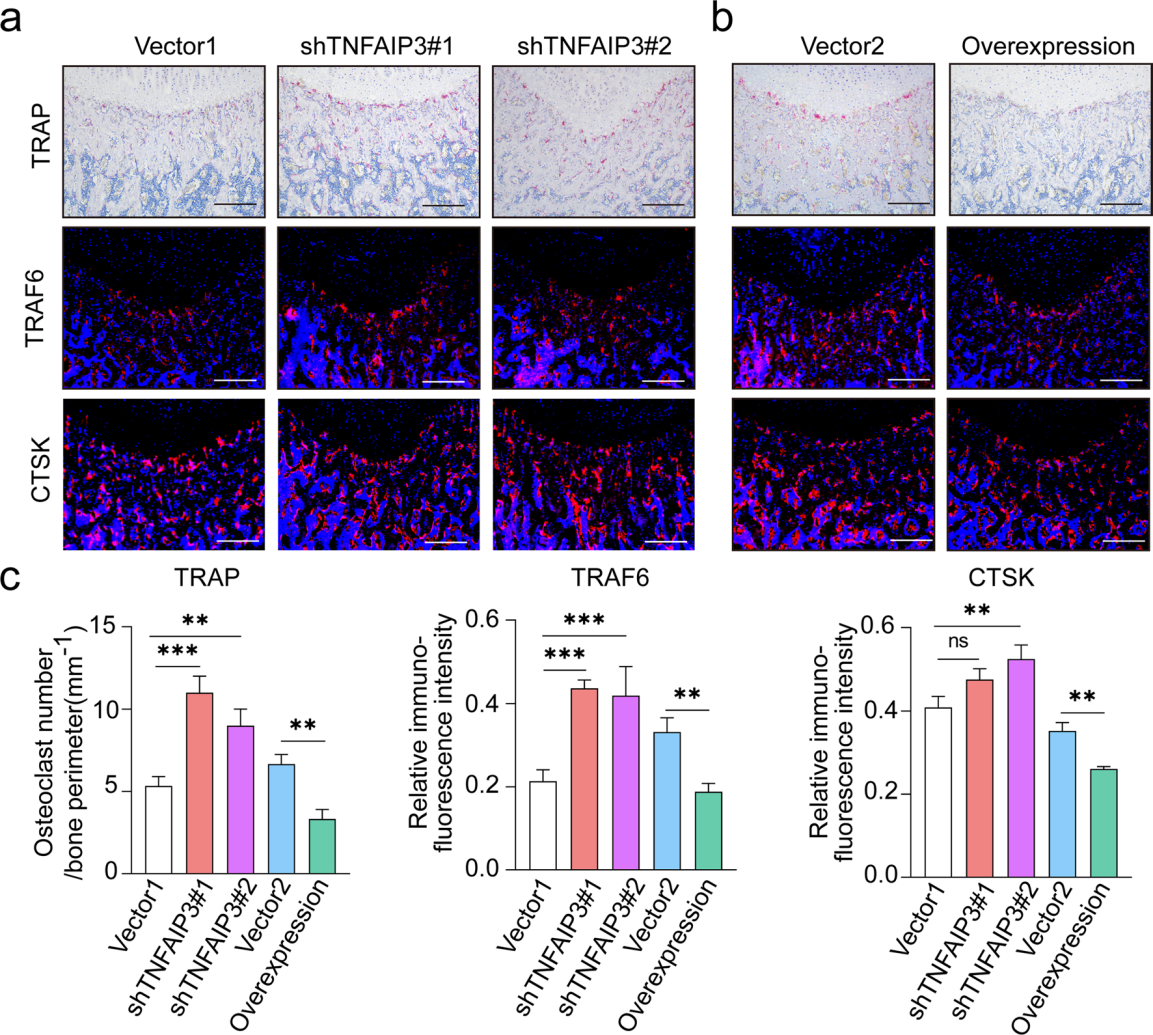
ACSC-specific TNFAIP3 overexpression inhibits osteoclastogenesis in vivo.** a, b** TRAP staining (top) and immunofluorescence of TRAF6 (middle) and CTSK (bottom) (genes associated with OC formation and OC differentiation) in the subchondral bone of OA rats showed that overexpression of TNFAIP3 in ACSCs inhibits osteoclast formation, while knockdown of TNFAIP3 in ACSCs impaired their inhibitory effects in vivo. Scale bars, 200 μm. **c** Quantification of osteoclast number (left) and the relative fluorescence intensity of TRAF6 (middle) and CTSK (right) in **(a**–**b)**. The statistical significance of differences was determined using one-way ANOVA with multiple comparison tests (LSD). Experiments were repeated independently for more than three times. All data are shown as the mean ± S.D. **P < 0.01, ***P < 0.001; ns, not significant

Next, to investigate the potential role of *TNFAIP3* gene modification on the in vivo therapeutic effects of ACSCs for alleviating subchondral bone lesions, TNFAIP3 over-expressing ACSCs or TNFAIP3 deficient ACSCs (Additional files 6, 7: Figure S3-4) were delivered to OA rat knees via intra-articular injection. The micro-CT data revealed that deficiency of TNFAIP3 lead to poor protection effects of ACSCs on the subchondral bone remodeling in OA rat knees (Fig. 7a). On the contrary, TNFAIP3 overexpression in ACSCs significantly improved subchondral bone structures and alleviated bone volume loss compared to the vector group (Fig. 7b). Additionally, TNFAIP3 over-expressing ACSCs yield better protective effects on the osteo-chondral tissue and alleviate the abnormal subchondral bone remodeling in OA rat knees than their counterpart vector group. In contrast, TNFAIP3 knocking-down in ACSCs impaired their protective effects on knee tissues and subchondral bone remodeling in vivo (Fig. 7c–f). The analysis of histology, immunohistochemistry staining, and Mankin scoring showed similar results (Fig. 7g–i). To further explore the effect of TNFAIP3 expression in ACSCs on the in situ osteoclast formation in OA rat knees, TRAP, TRAF6 and CTSK staining were performed. The pathological results showed that TNFAIP3 overexpression enhanced the inhibition of ACSCs on osteoclast formation in vivo while TNFAIP3 knocking-down partially mitigate the suppression of ACSCs (Fig. 8a–c).

## Discussion

In the current study, we describe an easy and reproducible method to harvest human ACSCs that does not require cell sorting or adhesion to fibronectin techniques. In addition, the ACSCs we generated exhibited significant protective effects on the osteo-chondral tissue of OA rats. Moreover, our findings demonstrate that OA microenvironments increase the secretion of TNFAIP3 which contributes to the improvement of subchondral bone remodeling by targeting osteoclasts (Additonal file 6).

Recently, Rikkers et al. summarizes the state-of-the-art research on the studies of cartilage derived stem cells including their isolation procedures [36]. Due to the lack of expression of fibronectin receptors and no unique cell marker or marker set has been established to specifically identify ACSCs, the isolation and characterization protocols vary greatly [36–41]. In the present study, we develop a reliable and user-friendly protocol for the isolation and culture of large numbers of ACSCs from human articular cartilages by advantage of the migratory properties of ACSCs. Previous studies have demonstrated that MSCs upregulate PDPN at sites of chronic inflammation and enhance the migratory capacity of tissue-resident MSCs. In the current study, ACSCs highly express PDPN, which may improve the cell yields [42]. In addition, the combination of injury induction, shaker-mediated incubation, enzyme-digestion to articular cartilage further improves the efficacy of isolation procedure and cell yields. Further functional assays including colony formation, multi-differentiation, and cell proliferation proved the stem cell properties of the ACSCs we harvested by using this protocol. However, the sub-populations in ACSCs and their functional heterogeneity need to be further clarified by performing single-cell scale analysis in future study.

Though cartilage derived stem cells have been proved to be involved in cartilage development, homostasis and regeneration, few information is available about their role on osteoclast formation. The RNA-seq data in our study showed that human ACSCs express numerous regulatory factors of osteoclasts including *RANKL*, *TNFRSF11B*, and *M-CSF*. Additionally, except for the classical *RANKL*/*RANK*/*TNFRSF11B* factors, ACSCs also express *TNFAIP3* and *TSG6*, which play pivotal roles in response to inflammatory stress. Moreover, we found that OASF derived TNF-ɑ promoted the release of TNFAIP3 from ACSCs. To the best of our knowledge, there is no previous studies of ACSC secreting TNFAIP3 and their role in osteoclast formation so far.

TNFAIP3 is both a ubiquitin-editing enzyme and an anti-inflammatory protein. It is a negative regulator of NF-κB that plays important roles in immune responses and programmed cell death, which make it an attractive target for the treatment of arthritis [43]. Hong et al. reported that TNFAIP3 was upregulated in neutrophils and gingival tissues of patients with periodontitis [44]. In addition, LPS- and nicotine pretreatment induced the expression of TNFAIP3 in human periodontal ligament cells. Moreover, Yan et al. further demonstrated that human periodontal ligament cells under hypoxia secreted more TNFAIP3 and inhibited osteoclast formation via targeting TRAF6 which stimulates osteoclast formation [45]. In the current study, TNFAIP3 overexpression decreased the size and number of osteoclasts and the expression of osteoclast formation genes while TNFAIP3 deficiency in ACSCs impaired the suppressive effects on osteoclasts. However, further explorations of the secondary effects on osteoclasts post-TNFAIP3 modification need to be performed in future studies. Most recently, Martens et al. reported that TNFAIP3 deficient mice derived osteoclast precursors were hyper-responsive to RANKL-induced osteoclast formation. Mechanistically, TNFAIP3 was recruited to the RANK receptor complex and restrained NF-κB activation via its zinc finger (ZnF) 4 and 7 ubiquitin-binding functions [46].

In the current study, the way of ACSC derived TNFAIP3 to subchondral bone remains to be elucidated. Previously, the calcified cartilage between joint cartilage and subchondral bone was believed to be impermeable for the transport of solutes. However, numerous studies demonstrated that small molecules could penetrate the calcified cartilage [47, 48]. In addition, the components of the osteochondral structure underwent marked alterations during the evolution of OA, which might help the perfusion of secreted factors from the  joint to the subchondral bone [45]. Further validation of imaging or biomarkers is needed to identify the underlying processes in future studies (Additional file 7).

We must acknowledge several limitations in our study. First, due to the lack of healthy human articular cartilage, the ACSCs used in the present study were harvested from OA cartilage and the pathological changes in cartilaginous tissues may exert potential influences on the cell properties of ACSCs, and the safeness of cytotherapy. Second, in the present study, the ACSCs were given to OA rats in PBS, and optimized cell delivery strategy may improve the therapeutic effects of ACSCs. Third, although OA is considered as a whole-joint disorder, we mainly focused on the suppressive effect of ACSCs and TNFAIP3-modified ACSCs on osteoclasts in our study, which does not elucidate the involvement of other cell types such as macrophages and osteoblasts which are also important in the pathogenesis of OA. Fourth, the in vivo distribution and the time for which intra-articularly injected stem cells stay within the knee joint need to be explored in future studies, which will improve the understanding of hACSCs-mediated protection for OA knees. Fifth, the concentration of TNF-ɑ within the OASF samples was partially influenced by patient selection because most of the cases are women and overweight based on their BMI, which probably causes a further increase of pro-inflammatory cytokines levels.

## Conclusion

In the present study, we reported the preparation and the protective effects of human ACSCs on osteo-chondral tissues in rat knee OA model. In addition, our findings revealed that osteoarthritic osteoclast is a novel cellular target of human ACSCs.

### Supplementary Information

**Additional file 1: Table S1.** Clinical and demographic characteristics of the study population undergoing TKA. The age range, body weight, gender and TKA information of eight patients in the current study has been included in Table S1.

**Additional file 2: Figure S1.** Characterization of ACSCs. **a** After 7 days of culturing, fibroblast-like cells migrate out from cartilage chips. **b** The result of ACSCs morphology at passage 4 and passage 8. **c** Flow cytometry staining demonstrated that negative express the hematopoietic and endothelial markers (CD45, CD31 and CD235A), and were positive for MSC markers (such as CD29, CD44, CD90, CD105 and PDPN) (Fig. S1c). The Scale bars represent 200μm (Fig. S1a, S1b), respectively. Experiments were repeated independently for more than three times. All data are shown as the mean ± S.D.

**Additional file 3: Table S2.** Demographic, clinical, and imaging characteristics of the OASF sample population. The age range, gender, BMI, symptom duration, and MOAKS synovitis/effusion of the OASF donors have been included in Table S2.

**Additional file 4: Figure S2.** The population doubling time (PDT) of hACSCs from P1 to P5.

**Additional file 5: Table S3.** Concentration of TNF-ɑ within the OASF before and after neutralization (pg/mL). The concentration of TNF-ɑ within the OASF before and after neutralization (pg/mL) has been included in Table S3.

**Additional file 6: Figure S3.** The protein level of TNFAIP3 in gene-modified hACSCs detected by WB.

**Additional file 7: Figure S4.** The original western blot gel for Figure S3.

## Data Availability

The raw sequence reads of RNA-seq data reported in this paper have been deposited at the Sequence Read Archive (SRA) database under the accession number PRJNA983968 and are publicly accessible with either https://www.ncbi.nlm.nih.gov/sra/PRJNA983968 or https://www.ncbi.nlm.nih.gov/Traces/study/?acc=PRJNA983968&o=acc_s%3Aa. The Submission ID is SUB13524596.

## Abbreviations

ACSCs: Articular cartilage stem cells
OA: Osteoarthritis
TNFAIP3: TNF alpha-induced protein 3
MAPK: Mitogen-activated protein kinase
NF-κB: Nuclear factor-κB
NSAIDs: Nonsteroidal anti-inflammatory drugs
HA: Hyaluronic acid
MSC: Mesenchymal stem cell
TNF-ɑ: Tumor necrosis factor ɑ
PBS: Phosphate-buffered saline
α-MEM: α-Minimal essential Eagle’s medium
FBS: Fetal bovine serum
CFU-F: Colony forming unit-fibroblast
ALP: Alkaline phosphatase
PE: Phycoerythrin
PE–cy7: Phycoerythrin–cyanine7
FITC: Fluorescein isothiocyanate
APC: Allophycocyanin
PDPN: Podoplanin
PTOA: Post-traumatic osteoarthritis
ACLT: Anterior cruciate ligament transection
micro-CT: Microcomputed tomography
BV/TV: Bone volume fraction
Tb.Th: Trabecular bone thickness
Tb.N: Trabecular bone number
Tb.Sp: Trabecular separation
SOFG: Safranin O–fast green
TRAP: Tartrate-resistant acid phosphatase
TRAF6: TNF receptor-associated factor 6
CTSK: Cathepsin K
GO: Gene ontology
GSEA: Gene set enrichment analysis
ELISA: Enzyme-linked immunosorbent assay
PBMC: Peripheral blood mononuclear cell
M-CSF: Microphage colony stimulating factor
RANKL: Receptor activator of nuclear factor-kappa B ligand
OASF: Synovial fluid from OA patients
TNFRSF11B: TNF receptor superfamily member 11b
PDT: Population doubling time

## References

[1] Yu Y, Zheng H, Buckwalter JA, Martin JA (2014). Single cell sorting identifies progenitor cell population from full thickness bovine articular cartilage. Osteoarthr Cartil.

[2] Jiang Y, Tuan RS (2015). Origin and function of cartilage stem/progenitor cells in osteoarthritis. Nat Rev Rheumatol.

[3] Jiang Y, Cai Y, Zhang W, Yin Z, Hu C, Tong T (2016). Human cartilage-derived progenitor cells from committed chondrocytes for efficient cartilage repair and regeneration. Stem Cells Transl Med.

[4] Wu MJM, Sermer C, Kandel RA, Theodoropoulos JS (2022). Characterization of migratory cells from bioengineered bovine cartilage in a 3D co-culture model. Am J Sports Med.

[5] Wang K, Li J, Li Z, Wang B, Qin Y, Zhang N (2019). Chondrogenic progenitor cells exhibit superiority over mesenchymal stem cells and chondrocytes in platelet-rich plasma scaffold-based cartilage regeneration. Am J Sports Med.

[6] Xie X, Wang Y, Zhao C, Guo S, Liu S, Jia W (2012). Comparative evaluation of MSCs from bone marrow and adipose tissue seeded in PRP-derived scaffold for cartilage regeneration. Biomaterials.

[7] Koelling S, Kruegel J, Irmer M, Path JR, Sadowski B, Miro X (2009). Migratory chondrogenic progenitor cells from repair tissue during the later stages of human osteoarthritis. Cell Stem Cell.

[8] Zhou C, Zheng H, Buckwalter JA, Martin JA (2016). Enhanced phagocytic capacity endows chondrogenic progenitor cells with a novel scavenger function within injured cartilage. Osteoarthr Cartil.

[9] Sharma L (2021). Osteoarthritis of the knee. N Engl J Med.

[10] Hunter DJ, Bierma-Zeinstra S (2019). Osteoarthr Lancet.

[11] Arden NK, Perry TA, Bannuru RR, Bruyère O, Cooper C, Haugen IK, Hochberg MC, McAlindon TE, Mobasheri A, Reginster JY (2021). Non-surgical management of knee osteoarthritis: comparison of ESCEO and OARSI 2019 guidelines. Nat Rev Rheumatol.

[12] Katz JN, Arant KR, Loeser RF (2021). Diagnosis and treatment of hip and knee osteoarthritis: a review. JAMA.

[13] Jiang W, Jin Y, Zhang S, Ding Y, Huo K, Yang J (2022). PGE2 activates EP4 in subchondral bone osteoclasts to regulate osteoarthritis. Bone Res.

[14] Tsukasaki M, Takayanagi H (2019). Osteoimmunology: evolving concepts in bone-immune interactions in health and disease. Nat Rev Immunol.

[15] Hu W, Chen Y, Dou C, Dong S (2021). Microenvironment in subchondral bone: predominant regulator for the treatment of osteoarthritis. Ann Rheum Dis.

[16] Pippenger BE, Duhr R, Muraro MG, Pagenstert GI, Hügle T, Geurts J (2015). Multicolor flow cytometry-based cellular phenotyping identifies osteoprogenitors and inflammatory cells in the osteoarthritic subchondral bone marrow compartment. Osteoarthr Cartil.

[17] Duarte JH (2014). Osteoarthritis: alendronate treatment improves pathology in animal model of OA by blocking osteoclastic bone resorption. Nat Rev Rheumatol.

[18] Bannuru RR, Osani MC, Vaysbrot EE (2019). OARSI guidelines for the non-surgical management of knee, hip, and polyarticular osteoarthritis. Osteoarthr Cartil.

[19] Ng J, Little CB, Woods S, Whittle S, Lee FY, Gronthos S (2020). Stem cell-directed therapies for osteoarthritis: the promise and the practice. Stem Cells.

[20] Zelinka A, Roelofs AJ, Kandel RA, De Bari C. Cellular therapy and tissue engineering for cartilage repair. Osteoarthritis Cartilage. 2022;20:S1063–4584(22)00855-X. 10.1016/j.joca.2022.07.012 36150678

[21] Le Blanc K, Mougiakakos D (2012). Multipotent mesenchymal stromal cells and the innate immune system. Nat Rev Immunol.

[22] Sui BD, Hu CH, Liu AQ, Zheng CX, Xuan K, Jin Y (2019). Stem cell-based bone regeneration in diseased microenvironments: challenges and solutions. Biomaterials.

[23] Berenbaum F, Wallace IJ, Lieberman DE, Felson DT (2018). Modern-day environmental factors in the pathogenesis of osteoarthritis. Nat Rev Rheumatol.

[24] Shang F, Yu Y, Liu S, Ming L, Zhang Y, Zhou Z (2020). Advancing application of mesenchymal stem cell-based bone tissue regeneration. Bioact Mater.

[25] Xu C, Feng C, Huang P, Li Y, Liu R, Liu C (2022). TNFα and IFNγ rapidly activate PI3K-AKT signaling to drive glycolysis that confers mesenchymal stem cells enhanced anti-inflammatory property. Stem Cell Res Ther.

[26] Ren G, Zhang L, Zhao X, Xu G, Zhang Y, Roberts AI (2008). Mesenchymal stem cell-mediated immunosuppression occurs via concerted action of chemokines and nitric oxide. Cell Stem Cell.

[27] He J, Yan J, Wang J, Zhao L, Xin Q, Zeng Y (2021). Dissecting human embryonic skeletal stem cell ontogeny by single-cell transcriptomic and functional analyses. Cell Res.

[28] Wang YX, Zhao ZD, Wang Q, Li ZL, Huang Y, Zhao S (2020). Biological potential alterations of migratory chondrogenic progenitor cells during knee osteoarthritic progression. Arthritis Res Ther.

[29] Li X, Ding L, Wang YX, Li ZL, Wang Q, Zhao ZD (2020). Skeletal stem cell-mediated suppression on inflammatory osteoclastogenesis occurs via concerted action of cell adhesion molecules and osteoprotegerin. Stem Cells Transl Med.

[30] Zhu H, Yang F, Tang B, Li XM, Chu YN, Liu YL (2015). Mesenchymal stem cells attenuated PLGA-induced inflammatory responses by inhibiting host DC maturation and function. Biomaterials.

[31] Zhu H, Guo ZK, Jiang XX, Li H, Wang XY, Yao HY (2010). A protocol for isolation and culture of mesenchymal stem cells from mouse compact bone. Nat Protoc.

[32] Li PL, Wang YX, Zhao ZD, Li ZL, Liang JW, Wang Q (2021). Clinical-grade human dental pulp stem cells suppressed the activation of osteoarthritic macrophages and attenuated cartilaginous damage in a rabbit osteoarthritis model. Stem Cell Res Ther.

[33] Ofner D, Hittmair A, Marth C (1992). Relationship between quantity of silver stained nucleolar organizer regions associated proteins (Ag-NORs) and population doubling time in ten breast cancer cell lines. Pathol Res Pract.

[34] Kim JE, Lee SM, Kim SH, Tatman P, Gee AO, Kim DH, Lee KE, Jung Y, Kim SJ (2014). Effect of self-assembled peptide-mesenchymal stem cell complex on the progression of osteoarthritis in a rat model. Int J Nanomed.

[35] Jensen EC (2013). Quantitative analysis of histological staining and fluorescence using ImageJ. Anat Rec.

[36] Rikkers M, Korpershoek JV, Levato R, Malda J, Vonk LA (2022). The clinical potential of articular cartilage-derived progenitor cells: a systematic review. NPJ Regen Med.

[37] Tao T, Li Y, Gui C, Ma Y, Ge Y, Dai H (2018). Fibronectin enhances cartilage repair by activating progenitor cells through integrin α5β1 receptor. Tissue Eng Part A.

[38] Mantripragada VP, Bova WA, Boehm C, Piuzzi NS, Obuchowski NA, Midura RJ (2018). Primary cells isolated from human knee cartilage reveal decreased prevalence of progenitor cells but comparable biological potential during osteoarthritic disease progression. J Bone Jt Surg Am.

[39] Su X, Zuo W, Wu Z, Chen J, Wu N, Ma P (2015). CD146 as a new marker for an increased chondroprogenitor cell sub-population in the later stages of osteoarthritis. J Orthop Res.

[40] Zhou C, Zheng H, Seol D, Yu Y, Martin JA (2014). Gene expression profiles reveal that chondrogenic progenitor cells and synovial cells are closely related. J Orthop Res.

[41] Pretzel D, Linss S, Rochler S, Endres M, Kaps C, Alsalameh S (2011). Relative percentage and zonal distribution of mesenchymal progenitor cells in human osteoarthritic and normal cartilage. Arthritis Res Ther.

[42] Ward LSC, Sheriff L, Marshall JL, Manning JE, Brill A, Nash GB, McGettrick HM (2019). Podoplanin regulates the migration of mesenchymal stromal cells and their interaction with platelets. J Cell Sci.

[43] Wu Y, He X, Huang N, Yu J, Shao B (2020). A20: a master regulator of arthritis. Arthritis Res Ther.

[44] Hong JY, Bae WJ, Yi JK, Kim GT, Kim EC (2016). Anti-inflammatory and anti-osteoclastogenic effects of zinc finger protein TNFAIP3 overexpression in human periodontal ligament cells. J Periodontal Res.

[45] Yan K, Wu C, Ye Y, Li L, Wang X, He W (2020). TNFAIP3 inhibits osteoclastogenesis via TRAF6-dependent autophagy in human periodontal ligament cells under hypoxia. Cell Prolif.

[46] Martens A, Hertens P, Priem D, Rinotas V, Meletakos T, Gennadi M (2022). TNFAIP3 controls RANK-dependent osteoclast formation and bone physiology. EMBO Rep.

[47] Goldring SR, Goldring MB (2016). Changes in the osteochondral unit during osteoarthritis: structure, function and cartilage-bone crosstalk. Nat Rev Rheumatol.

[48] Pan J, Zhou XZ, Li W, Novotny JE, Doty SB, Wang LY (2009). In situ measurement of transport between subchondral bone and articular cartilage. J Orthop Res.

