# Supplementary material for: Human osteoarthritic articular cartilage stem cells suppress osteoclasts and improve subchondral bone remodeling in experimental knee osteoarthritis partially by releasing TNFAIP3
Source: Stem Cell Res Ther. 2023 Sep 27;14:253. doi: 10.1186/s13287-023-03411-7 (PMC10523665; doi:10.1186/s13287-023-03411-7)
Supplement: Supplementary file 1 — Additional file 1: Table S1. Clinical and demographic characteristics of the study population undergoing TKA. The age range, body weight, gender and TKA information of eight patients in the current study has been included in Table S1. [file 13287_2023_3411_MOESM1_ESM.docx]

Table S1. Clinical and demographic characteristics of the study population undergoing TKA

| Patient ID | Age (years) | Weight  (Kg) | Gender | TKA |
| --- | --- | --- | --- | --- |
| OA1 | 60-69 | 69 | M | Left |
| OA2 | 70-79 | 60 | M | Right |
| OA3 | 50-59 | 75 | F | Left |
| OA4 | 70-79 | 58 | F | Right |
| OA5 | 60-69 | 64 | F | Right |
| OA6 | 60-69 | 68 | F | Right |
| OA7 | 60-69 | 70 | F | Right |
| OA8 | 60-69 | 64 | F | Right |

M, Male; F, Female; TKA: total knee arthroplasty
