# Supplementary figures and images for: Human osteoarthritic articular cartilage stem cells suppress osteoclasts and improve subchondral bone remodeling in experimental knee osteoarthritis partially by releasing TNFAIP3
Source: Stem Cell Res Ther. 2023 Sep 27;14:253. doi: 10.1186/s13287-023-03411-7 (PMC10523665; doi:10.1186/s13287-023-03411-7)

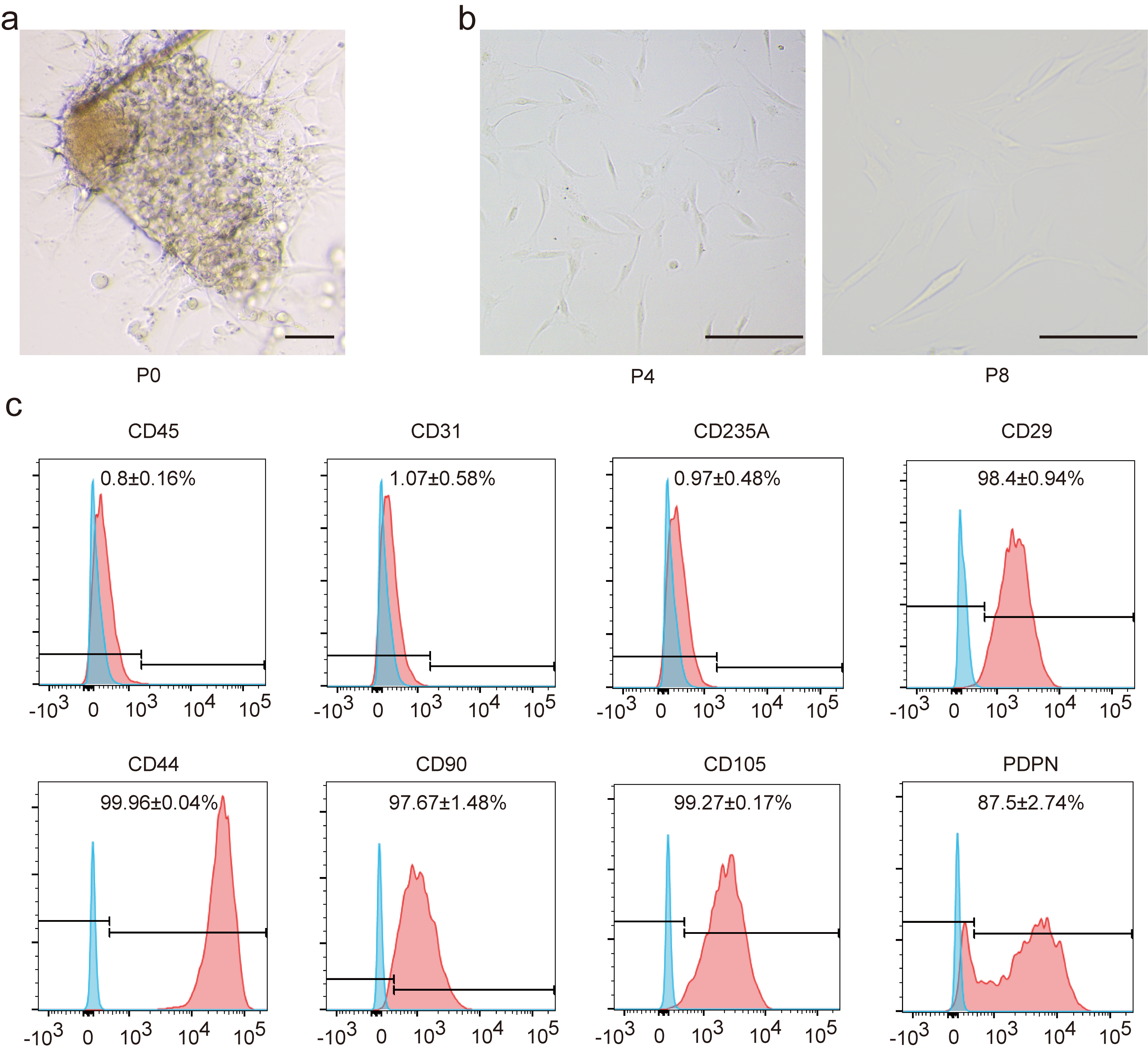

Supplement: Supplementary file 2 — Additional file 2: Figure S1. Characterization of ACSCs. a After 7 days of culturing, fibroblast-like cells migrate out from cartilage chips. b The result of ACSCs morphology at passage 4 and passage 8. c Flow cytometry staining demonstrated that negative express the hematopoietic and endothelial markers (CD45, CD31 and CD235A), and were positive for MSC markers (such as CD29, CD44, CD90, CD105 and PDPN) (Fig. S1c). The Scale bars represent 200μm (Fig. S1a, S1b), respectively. Experiments were repeated independently for more than three times. All data are shown as the mean ± S.D. [file 13287_2023_3411_MOESM2_ESM.tif]

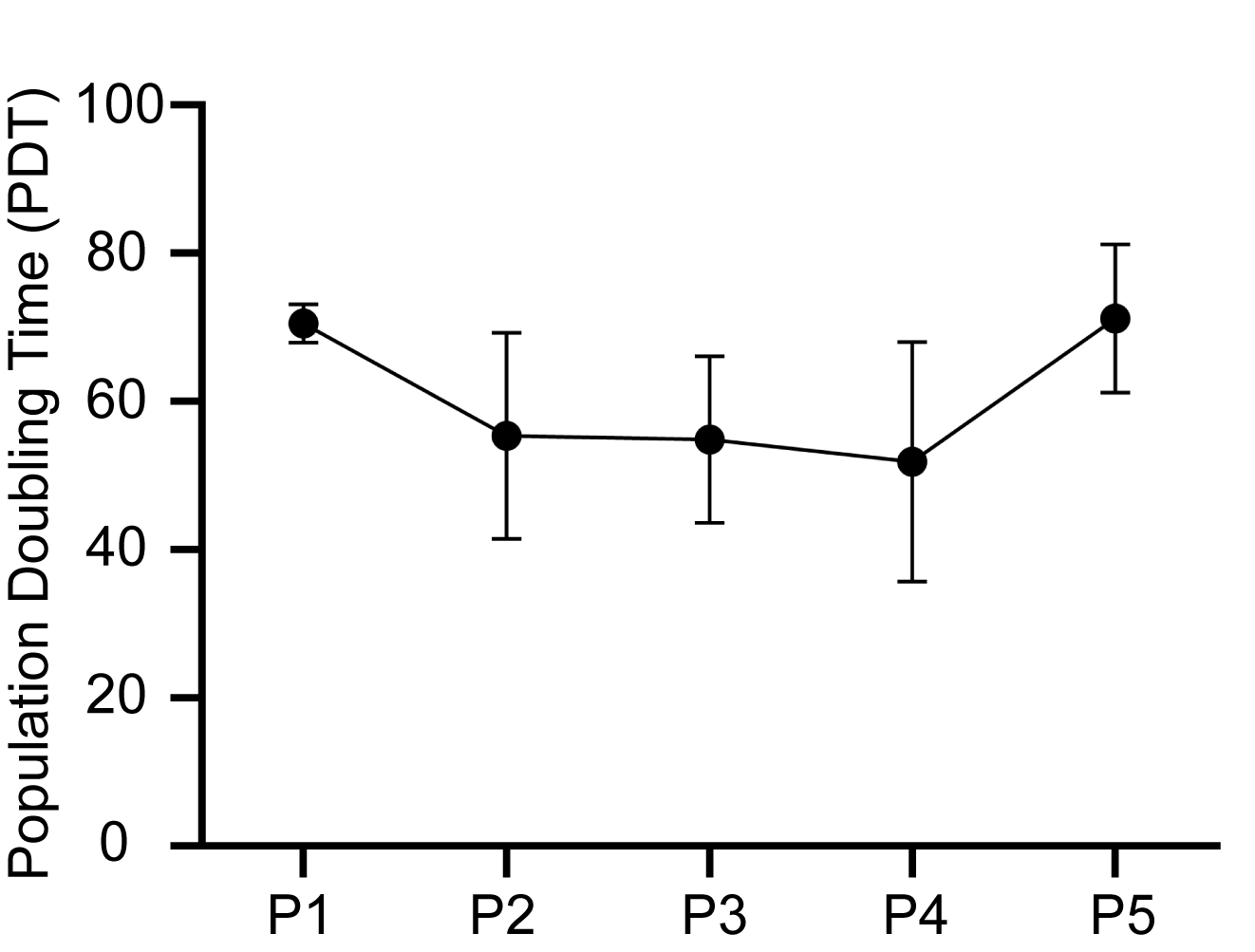

Supplement: Supplementary file 4 — Additional file 4: Figure S2. The population doubling time (PDT) of hACSCs from P1 to P5. [file 13287_2023_3411_MOESM4_ESM.tif]

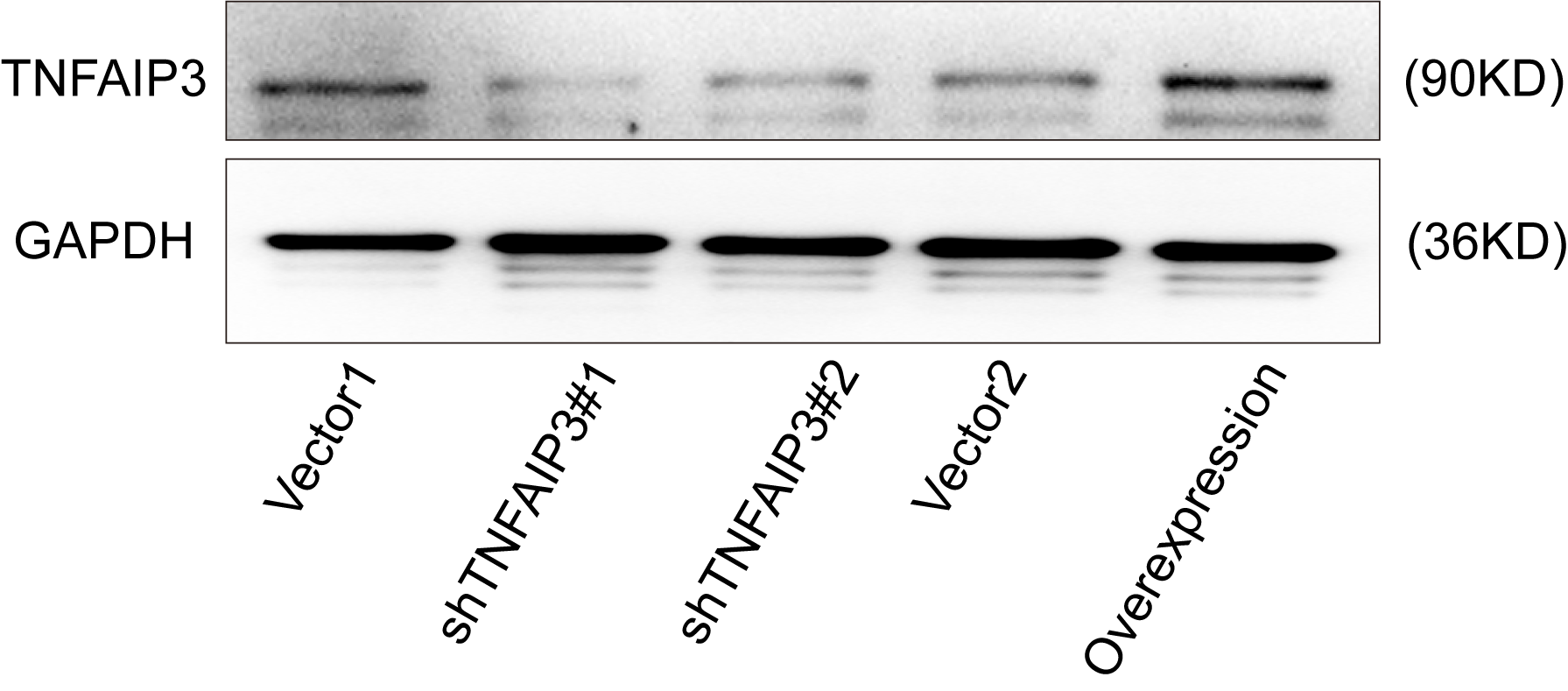

Supplement: Supplementary file 6 — Additional file 6: Figure S3. The protein level of TNFAIP3 in gene-modified hACSCs detected by WB. [file 13287_2023_3411_MOESM6_ESM.tif]

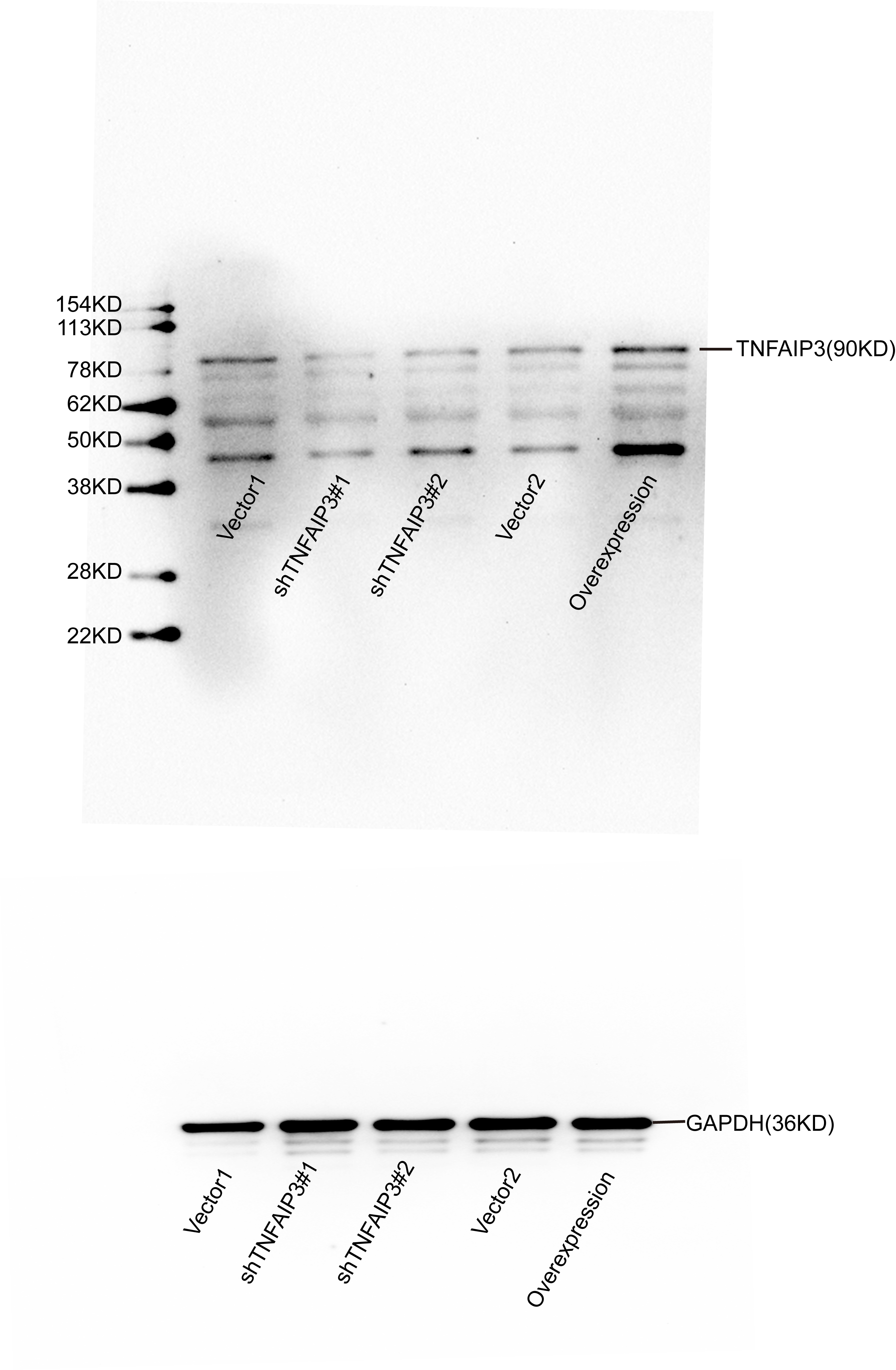

Supplement: Supplementary file 7 — Additional file 7: Figure S4. The original western blot gel for Figure S3. [file 13287_2023_3411_MOESM7_ESM.tif]
