# Supplementary material for: Human osteoarthritic articular cartilage stem cells suppress osteoclasts and improve subchondral bone remodeling in experimental knee osteoarthritis partially by releasing TNFAIP3
Source: Stem Cell Res Ther. 2023 Sep 27;14:253. doi: 10.1186/s13287-023-03411-7 (PMC10523665; doi:10.1186/s13287-023-03411-7)
Supplement: Supplementary file 3 — Additional file 3: Table S2. Demographic, clinical, and imaging characteristics of the OASF sample population. The age range, gender, BMI, symptom duration, and MOAKS synovitis/effusion of the OASF donors have been included in Table S2. [file 13287_2023_3411_MOESM3_ESM.docx]

Table S2. Demographic, clinical, and imaging characteristics in OA patients for OASF collection

| No. of  patients | Age (y) | Gender | BMI (kg/m^2^) | Symptom duration (years) | MOAKS Synovitis/effusion |
| --- | --- | --- | --- | --- | --- |
| 1 | 70-79 | M | 24 | 8 | 3 |
| 2 | 60-69 | F | 25 | 9 | 2 |
| 3 | 70-79 | F | 26 | 11 | 3 |
| 4 | 60-69 | M | 24 | 9 | 2 |
| 5 | 50-59 | F | 25 | 3 | 2 |
| 6 | 50-59 | F | 25 | 4 | 2 |
| 7 | 50-59 | F | 30 | 8 | 2 |
| 8 | 50-59 | F | 23 | 5 | 2 |
| 9 | 50-59 | M | 24 | 4 | 2 |
| 10 | 50-59 | F | 27 | 7 | 2 |

OASF,Synovial fluid from OA patients; BMI, Body Mass Index; MOAKS, MRI Osteoarthritis Knee Score; M, Male; F, Female
