# Supplementary material for: Human osteoarthritic articular cartilage stem cells suppress osteoclasts and improve subchondral bone remodeling in experimental knee osteoarthritis partially by releasing TNFAIP3
Source: Stem Cell Res Ther. 2023 Sep 27;14:253. doi: 10.1186/s13287-023-03411-7 (PMC10523665; doi:10.1186/s13287-023-03411-7)
Supplement: Supplementary file 5 — Additional file 5: Table S3. Concentration of TNF-ɑ within the OASF before and after neutralization (pg/mL). The concentration of TNF-ɑ within the OASF before and after neutralization (pg/mL) has been included in Table S3. [file 13287_2023_3411_MOESM5_ESM.docx]

Table S3. Concentration of TNF-ɑ within the OASF before and after neutralization (pg/mL)

| Sample  Repeat | 1 | 2 | 3 | 4 | 5 | 6 | 7 |
| --- | --- | --- | --- | --- | --- | --- | --- |
| OASF1 | 53.63 | 80.51 | 60.54 | 62.37 | 97.17 | 112.67 | 62.32 |
| OASF2 | 72.40 | 58.24 | 73.65 | 70.58 | 62.42 | 93.14 | 67.79 |
| OASF3 | 84.25 | 73.41 | 80.47 | 105.14 | 78.79 | 66.45 | 108.16 |
| OASF1 +  anti-TNF-α Ntab | 77.01 | 63.71 | 70.91 | 70.96 | 80.56 | 64.20 | 75.19 |
| OASF2 +  anti-TNF-α Ntab | 102.02 | 69.38 | 54.74 | 66.50 | 66.40 | 83.15 | 61.94 |
| OASF3 +  anti-TNF-α Ntab | 71.73 | 104.61 | 73.03 | 67.75 | 85.27 | 93.19 | 125.44 |

OASF: Synovial fluid from OA patients

Ntab: Neutralizing antibody
